# Scaling in the recovery of urban transportation systems from massive events

**DOI:** 10.1038/s41598-020-59576-1

**Published:** 2020-02-17

**Authors:** Aleix Bassolas, Riccardo Gallotti, Fabio Lamanna, Maxime Lenormand, José J. Ramasco

**Affiliations:** 1 Instituto de Física Interdisciplinar y Sistemas Complejos IFISC (CSIC-UIB), Campus UIB, 07122 Palma de Mallorca, Spain; 2 Fondazione Bruno Kessler, Via Sommarive 18, 38123 Povo, TN Italy; 3 TETIS, Univ Montpellier, AgroParisTech, CIRAD, CNRS, INRAE, Montpellier, France; 4 School of Mathematical Sciences, Queen Mary University, Mile End Road, E1 4NS London, United Kingdom

**Keywords:** Statistical physics, thermodynamics and nonlinear dynamics, Complex networks, Applied mathematics

## Abstract

Public transportation is a fundamental infrastructure for life in cities. Although its capacity is prepared for daily demand, congestion may rise when huge crowds gather in demonstrations, concerts or sport events. In this work, we study the robustness of public transportation networks by means of a stylized model mimicking individual mobility through the system. We find scaling relations in the delay suffered by both event participants and other citizens doing their usual traveling in the background. The delay is a function of the number of participants and the event location. The model is solved analytically in lattices proving the existence of scaling relations and the connection of their exponents to the local dimension. Thereafter, extensive and systematic simulations in eight worldwide cities reveal that a newly proposed measure of local dimension explains the exponents found in the network recovery. Our methodology allows to dynamically probe the local dimensionality of a transportation network and identify the most vulnerable spots in cities for the celebration of massive events.

## Introduction

The recent boost in accessibility to data on human mobility^1^ and infrastructures facilitates, more than ever, the study of human movement and transportation systems^2^. Human mobility covers a wide range of scales as a reflection of several transportation modes, yet the increase of urban population makes the management of inter-urban infrastructures a critical subject. For many years now, the dichotomy between private on-road and public transportation has dominated urban mobility. However, in a society increasingly concerned about climate change, and, with road traffic accounting for more one fifth of the total emissions in the US^3^, fostering the use of public transportation is nowadays a priority. Among the factors driving the public transportation use^4,5^, improving travel times and keeping congestion under control are fundamental.

In recent years, complex network theory has been proved as a useful tool to model and understand dynamics of real world systems like epidemic spreading^6^, transportation systems^7^ or social interactions^8^. Transportation systems (e.g., road, rail, bus, air) can be naturally mapped into networks, which helps to unveil systemic features^9,10^. Similarly to power grids, transportation networks are intrinsically spatial^11,12^, space is inseparable from their topological and dynamical properties. The first works on public transportation focused mainly on their topology^13,14^, irrespective of the travel times between nodes and the spatial component. The next step was to introduce weights as a representation of travel times or distances between nodes^15,16^. Those weights become even more relevant when more than one mode–with different characteristics–are intertwined, as in the case of public transportation^17,18^. The recent introduction of the multilayer framework^19,20^ and its development^21^ offer new tools to deal with a more complex reality, providing a unique perspective to model multimodal transportation. From a theoretical point of view, it was shown that multilayer networks can, by themselves, give rise to congestion^22^ and that an increase of link speeds or capacities does not necessarily improve network performance^23^. The onset of congestion in multiplexes (multilayer networks in which each node represents the same entity in different layers) have been studied as a function of the number of layers and degenerate paths^24^. From a practical perspective, many works have been published studying multimodal transportation networks as multiplexes: From works where every transportation mode is a single layer^17,25,26^ to others using a layer for each line^18,27^.

Understanding the emergence of congestion and delays requires, however, a step beyond the static structure of the network. First of all, in both private and public transportation the capacities of lines, roads and vehicles are of wide variety, and need to be taken into account to evaluate congestion and vulnerability^23,28,29^. Secondly, the transportation demand in cities is by no means homogeneous. Neither the origins and destinations of trips are equiprobable nor the number of travelers is constant in time. In fact, the interest of planners in the spatio-temporal characteristics of demand is almost as old as transportation research^30^. Luckily, nowadays we have new sources of data to understand urban mobility as a cheaper alternative to the usual surveys^31–33^. While most works focus on shortest paths to infer the routing of individuals, the way citizens choose their route is far from trivial^34,35^, specially in the congested phase in which their routes might be readapted^36^.

Whereas transportation infrastructures are prepared for the daily demand, they might fail to manage shocks and disruptions^17,37^. Particularly, concerts, sport events or massive demonstrations are recurrent in metropolises, gathering huge crowds in localized places. Such a peak of demand in a small area affects urban life as well as the public transportation system, yet this situation has attracted limited interest so far^38–41^. In this work, we examine how the delay of individuals leaving an event and that induced in the general public in the “background” depends on where and when it takes place and the number of attendees as the transportation network of the city recovers. Such dependence turns out to follow power-law-like (scaling) relations. The average delay in public transportation networks of eight worldwide cities (Amsterdam, Berlin, Boston, Madrid, Milan, New York City, Paris and San Francisco) has been numerically analyzed, showing that the scaling holds and the exponents can be explained by a newly proposed local dimension metric taking the capacity of lines into account. We prove beforehand the relation between the scaling and the network dimension by solving the model analytically in regular lattices.

## Methods

### Model description

Inspired by previous works on information transmission in computer systems^42^ and realistic agent-based models of traffic dynamics, we build a model to simulate how individuals move through a public transportation system using vehicles with limited capacity. The main ingredients are (*i*) the multilayer network, (*ii*) vehicle mobility, (*iii*) individuals trip planning and execution, and (*iv*) the distribution of origins and destinations of individuals (transportation demand).

The public transportation system of cities is, in general, formed by different modes, each one with its own characteristics in terms of speed, period, and capacity. The line period (or headway) is defined as the lapse of time between consecutive departures of vehicles in the same route. Similarly to^18,27^, we model the public transportation system as a multilayer network where every direction of each line is represented by one layer. The urban space is divided into a grid of cells, with size $400\,m\times 400\,m$. The stops of every line are assigned to the cell where they are located, so that the nodes of a layer correspond to cells hosting stops of the considered directed line. If more than one stop of the same line falls in the same cell, they are merged into a single node. Directional weighted intra-layer links are then created between nodes hosting consecutive stops. The weight corresponds to the travel time, calculated as the distance between connected cells centroids divided by the average speed of the transportation mode. Within this framework, all nodes have one incoming and one outgoing link except those containing the beginning and end of the line. In the multilayer network, we call a *location* the set of nodes associated to the same cell, and all nodes within the same location are connected through inter-layer links. Individuals can change line along these links with an average (walking) time penalty of 30 seconds. This quantity reflects the average time needed to move from one station to another but it can vary between modes. In the case of bus stops, which are a majority, vehicles usually stop at the same place so the time needed for the change is zero. For metro lines, the time might be greater than 30 seconds since it might imply the displacement from one platform to another. Note that the waiting time from the next vehicle is not included in this inter-layer penalty because it is explicitly modeled with the vehicle movements. In order to make sure that other penalty values do not significantly alter the scaling exponents, we have run simulations with a penalty of 120 seconds to replicate the results of the main paper (see Supplementary Note “Robustness of the results” and Supplementary Fig. S3). Vehicles leave the line head location with a fixed period and they go through all route nodes until the terminal. Since line periods vary throughout the day, when simulating cities we select the best case scenario by adopting the schedule of 8 am local time (rush hour). Note that in a more realistic situation, transportation managers may reinforce lines connecting to an event location as a planed response to decongest the system. We do not have detailed information on this type of preventive measures so we neglect them. More details on the transportation networks studied, schedules and geography can be found in Supplementary Table S1.

**Figure 1:**
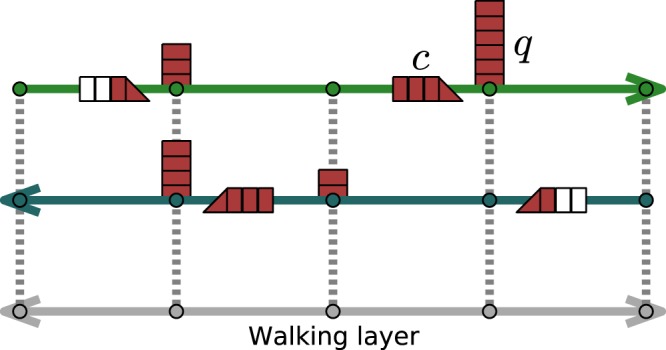
Sketch of the multilayer structure of our model. The two top layers are the transportation lines through which vehicles move. Individuals wait at each node generating a queue *q* until a vehicle arrives and they are able to board if the vehicle is below full capacity *c*. Below the transportation lines, in gray, a the walking layer with infinite capacity connected to the transportation lines layer through inter-layer links.

Besides all lines, we introduce a further walking layer with infinite capacity so that every cell is also a node in this layer. The nodes of the walking layer are connected within a certain radius ensuring its full connectivity, and travel times are calculated as the Haversine distance divided by a walking speed of 5 km/h. A sketch of how both directions of a line would be connected within our framework is depicted in Fig. 1. For simplicity and visualization purposes, the figure depicts a 1D lattice.

The actions of individuals have two phases: one of planning and one of execution. As the model is adaptive, these phases can be repeated until she/he arrives at the final destination. During the trip execution, individuals can move freely through the walking layer but need to use a vehicle to travel along links in the transportation lines. In case of line change, the waiting time is determined by the vehicle movements. At every node, we consider a queue *q* of individuals waiting for a vehicle. Individuals enter the queue and board in order (regulated on a “*first come, first served*” (FIFO) basis) until filling the vehicle or emptying the queue. For simulation convenience, if two individuals arrive at the stop at the same time the one that started the trip earlier has priority in the queue. Each vehicle has a capacity *c* given by the transportation mode and it is taken equal for all the vehicles of the line. Details on the capacities can be found in Supplementary Note “Dataset description”.

The routing protocol used by individuals for planning is adaptive with local information. In the absence of congestion, individuals follow the temporal optimal path of the static multilayer network calculated by the Dijkstra algorithm^43^. If there are line changes, they estimate, besides the change walking penalty, an additional waiting time of half the new line period (the real waiting time will be given by the vehicles location in the line when the individual arrives at the stop). Individuals’ route is only recalculated when a congested node, whose queue is larger than the vehicle’s capacity, is reached. In the case of rerouting, the link travel-time for an individual *i* attempting to board is updated with the expected waiting time given by

1 $${t}_{wait,i}=(\frac{1}{2}+[\frac{{q}_{i}}{c}])\,f,$$

where ${q}_{i}$ is the queue observed by individual *i*, *c* is the capacity of the line and *f* is the period, i.e. the time between consecutive vehicles. The square brackets $[{q}_{i}/c]$ represent the integer part of the ratio. Simply put, the new inter-layer weight will be the period multiplied by the number of vehicles necessary to empty the queue plus the normal waiting time of $f\mathrm{/2}$.

Throughout the work, individuals are separated in two groups: those participating in the event and those in the background. The first group is formed by *I* individuals introduced in the system at the place and time in which the massive event occurs. We assume that after it finishes they attempt to go home, so the destination of their trips is extracted from the home side of an Origin-Destination (OD) matrix characterizing the trip demand in each city/lattice (see below for details on the OD matrices). Event individuals are tracked until they reach their destination. Individuals in the background, on the other hand, are people who need to perform their daily trips. The origin of their trips is randomly selected respecting the home distribution of the OD matrix and their destination following the working one. New background individuals are introduced into the system at a rate of $\rho$ individuals per unit of time. Once a new background individual has an origin/destination assigned, he/she remains in the system until the trip completion. We ensure that $\rho$ is low enough so that delays are not generated in the standard operation scenario. More concretely and as a matter of practicality, we ensure that the most congested line is below (but close) to half its capacity. Several other values of $\rho$ have been explored and, in particular, simulations have been run without background, $\rho =0$, both in lattices and cities. The detail on the concrete values can be found in Supplementary Note “Dataset description”.

Summarizing, a simulation consists in generating background individuals and following their trips until the system attains a stationary state in terms of the load of active individuals. The background individuals continue entering in the system during the full simulation. Over this situation or in an empty network, if there is no background, *I* individuals are introduced in the place of the event and their trajectories are followed until the last of them reaches home. Several metrics are monitored in the system: first, separately for both background and event individuals the average delay $\Delta {\tau }_{av}$ calculated as the difference between the simulated time to perform the trip from origin to destination ${\tau }_{real}$ minus the expected time needed if no other individual would be in the network (over the optimal path) ${\tau }_{op}$. Besides individuals, we also measure the cumulative number of locations in which the queue of a stop has gone over its mode capacity (number of congested nodes or locations ${Q}_{T}$). These are the main metrics that we study as function of $I$ and of the event location.

### Data inputs

In the case of cities and to be realistic, the model needs data on the transportation network and on the trip demand. The transportation networks were obtained from Transitfeeds (https://transitfeeds.com), which contains information on the lines, stop locations, frequencies and schedules and travel times for the public transportation network of eight cities (see further details in the Supplementary Note on “Dataset description” and Supplementary Table S1). In the case of vehicle capacities, we needed to simplify given the possible variability, so they are fixed at 125 persons for the buses, 250 for tramway, 800 for the subway and 1000 for rail. In the case of San Francisco, the mini metro has a capacity of 125 persons and the cable cars of 70 persons.

Regarding the transportation demand, we have used geolocated Twitter data from 2014 to 2017 to estimate it. The data is obtained via the Twitter streaming API (https://developer.twitter.com/en/docs). The supplementary Note on “Dataset description” contains a template of the code needed to collect this data. As done in^31,44,45^, cities are divided in cells of $2\,km\times 2\,km$. The users location is obtained through their tweets: The most frequented cell from 8am to 8pm local time in working days is considered as work location, and as home location the most frequented cell in the complementary time period (See Supplementary Table S2). Tweets on Saturdays and Sundays, users moving faster than $300\,km/h$, tweeting more than ten times per minute, less than ten times in the whole time window and for less than one month have been disregarded. This method allows us to estimate commuting trip flows between every pair of cells in the cities. Twitter users are, of course, a fraction of the population so the Origin-Destination matrices correspond only to a sampling of the total. Still, since we are only interested in the transportation demand spatial distribution within each city, not in the final trip numbers, a normalization factor can be applied in every urban area dividing by the total number of local commuters.

In the case of the lattices, the capacities are fixed and constant in every line (except for the final exercise, where they are modified with the distance to the event). The demand is obtained by randomly assign origin and destination to the individuals’ trips.

## Results

### Scaling in lattices

We start the analysis in a controlled environment, as are regular lattices, where it is possible to attain analytical results and to gain insights on the system behavior before passing to transportation networks in cities.

#### Event individuals

**Figure 2:**
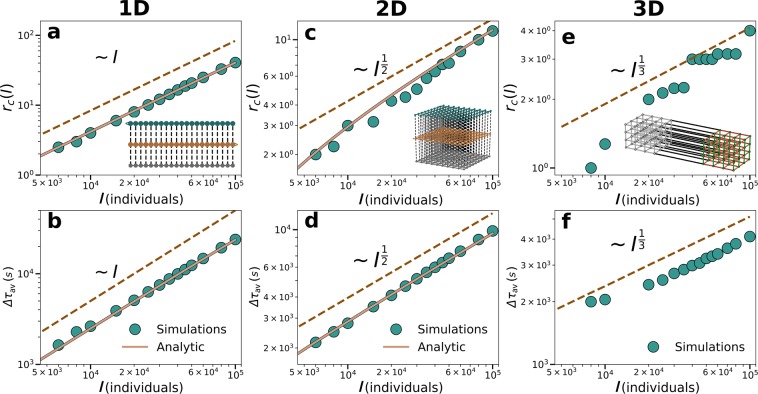
Results in lattices with the event located at the center. In 1D, simulations were performed in a lattice of size $N=L=1500$ nodes, capacity $c=600$ persons and period $f=600$ seconds; In 2D, with $N={L}^{2}={70}^{2}$ nodes, $c=80$ persons and $f=600$ seconds in all the lines; And, in 3D with $N={L}^{3}={25}^{3}$ nodes, $c=600$ persons and $f=600$ seconds. (**a**,**c**,**e**) radius of congestion ${r}_{c}$ depending on the attendees of the event $I$. (**b**,**d**,**f**) scaling of the average delay of agents depending on *I*. The dashed lines serve as a guide to the eye and the solid orange lines are the full analytical solutions. The solution for the radius of congestion can be found in Supplementary Eq. S11 (1D) and Eq. S29 (2D) and for the average delay in Eqs. S15 and S19 (1D), and Eqs. S31 and S34 (2D). All results correspond to the situation without background mobility and the inset shows a sketch of the multilayer network.

1D lattices: A transportation network (lattice) is built composed of only two lines going in opposite directions and a walking layer with infinite capacity connecting adjacent nodes (Fig. 1 and inset of Fig. 2a). In such situation, individuals have only two alternatives: waiting for the next vehicle or walking towards the next node. The optimal choice depends only on the queue at the current node and the parameters of the system. For simplicity, we focus first on the case without background $(\rho =0)$. Taking into account the travel time to the next node using a vehicle ${t}_{1}$ and by walking ${t}_{2}$, we can calculate from Eq. (1) the critical value of ${q}_{i}$ $({q}^{\ast })$ above which walking becomes the best option as

2 $${q}^{\ast }=[\frac{{t}_{2}-{t}_{1}}{f}+\frac{1}{2}]c\mathrm{}.$$

Recall that $\mathrm{[.]}$ stands for integer part and note that this equation is independent of the lattice dimension. Following the introduction of $I$ individuals in the event, ${q}^{\ast }$ of them stay at the origin node, and $I-{q}^{\ast }$ walk to the next node. As the wave of walking individuals move along the line, ${q}^{\ast }$ stay at every node until the number of remaining individuals reaching a node is below ${q}^{\ast }$. We calculate first the radius of congestion ${r}_{c}$, defined as the distance to the closest non congested stop in each direction. Given that event individuals split in two streams –left and right–, and that part of the individuals will reach their destination without ever entering a vehicle, ${r}_{c}(I)$ towards each direction can be obtained analytically (See Supplementary Note “1D analytical results”). In the limit of an infinite lattice $(L\to \infty )$, the analytical solution for ${r}_{c}(I)$ yields

3 $${r}_{c}(I)\sim \frac{I}{2\,{q}^{\ast }}\mathrm{}.$$

In Fig. 2a, we compare the analytical expression obtained for ${r}_{c}(I)$ with numerical simulations finding a good agreement between them.

From ${r}_{c}(I)$, we can analytically derive the average delay $\Delta {\tau }_{av}(I)$. We need to consider the two types of individuals: those that stay in each of the queues waiting for their turn, and those who walk until their final destination. In the first case, the delay is the sum of the time until all the previous nodes empty plus the time in emptying the local queue. In the second case, the delay is the distance between the event and their destination divided by the difference in speeds between walking and using a vehicle. The detailed analytical calculations, included in Supplementary Note “1D analytical results”, display a good agreement with the simulations as depicted in Fig. 2b. In the limit $L\to \infty$, our analytical solution approaches

4 $$\Delta {\tau }_{av}\sim \frac{f\,I}{4\,c}\mathrm{}.$$

The scaling in Fig. 2b applies to an event located at the center of the lattice, yet the analytical results hold for an event placed anywhere (Supplementary Fig. S1).

2D lattices: Empirical networks are closer to 2*D* lattices. The linear scaling obtained in 1D lattices is a product of the absence of alternatives: individuals must take the only existing line or walk to the next location. In higher dimensions, however, there is not only a degeneration of optimal paths (several isochronous paths lead to the same destination) but as they travel and find congested nodes many more alternatives become available. Moreover, those alternatives lead to a fragmentation of the flows in every intersection reaching faster a value under the vehicle capacity limit. 2*D* lattices of side *L* are composed of 4*L* layers containing one directed line and the additional walking layer. A sketch of the walking layer and two of the four directions is depicted in the inset of Fig. 2c. To estimate the extension of the congested area provided by ${r}_{c}(I)$ the relevant quantity is again ${q}^{\ast }$ (Eq. (2)). Since four lines (nodes) concur at every location and each node has an independent queue, more than ${q}^{\ast }$ individuals can be waiting at the same location. Considering that movements are directed and individuals are not allowed to return to previously visited nodes, not all directions will be selected. In fact, in 2D lattices, there will be three types of nodes: the node of the event that has 4 suitable directions, those in the main axes departing from it, which have 3 directions and the rest with only 2. For the sake of simplicity, we assume that the event occurs in the center of the lattice (or that the lattice is infinite) so that all directions are equivalent.

Unlike 1D lattices, now the number of suitable queues sitting at a distance *r*, ${N}_{q}(r)$, scales as ${N}_{q}(r)\sim {r}^{2}$. Dividing the space in equivalent quadrants and considering only individuals with destination within each quadrant $I{\prime} =\frac{I}{4}$, the results in 1D can be extended to calculate analytically the radius of congestion ${r}_{c}$ (See Supplementary Note “2D analytical results”). In the limit of $L\to \infty$, it goes as

5 $${r}_{c}\approx \sqrt{\frac{I{\prime} }{{q}^{\ast }}}\sim {(\frac{I}{4{q}^{\ast }})}^{\mathrm{1/2}}\mathrm{}.$$

While in the 1D lattice the radius of congestion was linear with *I*, in the 2D case it scales as ${I}^{\frac{1}{2}}$. The analytical results are confirmed by the simulations as depicted in Fig. 2c. The average delay suffered by the individuals in the event can then be calculated separating again between those waiting at the queues and those reaching their destination by walking. The complete analytical derivation is shown in Supplementary Note “2D analytical results” of the SI and in the limit $L\to \infty$ it yields

6 $$\Delta {\tau }_{av}\sim \frac{f\,{q}^{{\ast }^{2}}}{c}{I}^{\frac{1}{2}}\mathrm{}.$$

The results are displayed in Fig. 2d, confirming the agreement between the analytical solution and the simulations. The dashed line, with an exponent $\frac{1}{2}$, serves as guide to the eye.

General dimensions: For a general dimension $D > 2$, the scaling in the large $L$ and $I$ limits can be obtained with an approximation. Assuming that ${q}^{\ast }$ individuals remain at each node, we can estimate first the number of nodes congested as ${Q}_{T}\approx \frac{I}{{q}^{\ast }}$. In the limit $L\to \infty$, they will occupy an hypervolume of the order of $\sim {r}_{c}^{D}$ so that the radius of the affected area can be approximated as

7 $${r}_{c}\approx {(\frac{I}{{q}^{\ast }})}^{\mathrm{1/}D}\mathrm{}.$$

If the total delay is dominated by the individuals waiting for a vehicle as occurs in lower dimensions and using the radius of congestion, $\Delta {\tau }_{tot}$ can be approximated as

8 $$\Delta {\tau }_{tot}\approx \frac{{q}^{{\ast }^{2}}f}{c}\mathop{\sum }\limits_{r\mathrm{=0}}^{{r}_{c}-1}{N}_{q}(r),$$

where ${N}_{q}(r)$ is number of suitable queues that increases with the distance from the center of the lattice. The previous expression can be explained by the ${q}^{\ast }$ individuals that stay at each node and the delay they incur $f\,{q}^{\ast }/c$, which is the time that each node takes to empty. Considering that ${\sum }_{r\mathrm{=0}}^{{r}_{c}-1}{N}_{q}(r)\sim {r}_{c}^{D+1}\sim {I}^{\frac{D+1}{D}}$ and dividing by $I$, we find that

9 $$\Delta {\tau }_{av}\sim {I}^{\mathrm{1/}D}\mathrm{}.$$

In Fig. 2e,f, we check by means of simulations that the scaling exponent of $\mathrm{1/}D$ also holds in 3D lattices, showing how both ${r}_{c}$ and $\Delta {\tau }_{av}$ scale as ${I}^{\frac{1}{3}}$.

#### Effect of the background on the event participants

When the background is not zero, there is people traveling in the transportation network all the time. Given the trip demand, it is possible to calculate the load that every line needs to support. Such load subtracts capacity from the transportation mode considered. For example, if a subway vehicle capacity is 800 and there are already 200 users inside traveling further than the next stop, the effective capacity at the coming station is 600. This means that we can define an effective capacity ${c}_{eff}(i,m)$ depends on the particular location *i* and the transportation mode *m* in the stationary state of the background.

In lattices, since the transportation demand is homogeneous and there is only a transportation mode, the load supported by each line is related directly to the line (edge) betweenness calculated out of all the optimal paths between every pair of locations. This gives us an estimation for each $\rho$ of the value of ${c}_{eff}(i)$. Turning to a 1D lattice, the effective capacity in a location $x$ can be written as

10 $${c}_{eff}^{l}(x)=c-\rho \,f\frac{x(L-x)}{L(L-\mathrm{1)}}$$

for the links going to the left, while for those to the right by

11 $${c}_{eff}^{r}(x)=c-\rho \,f\frac{(x+\mathrm{1)(}L-1-x)}{L(L-\mathrm{1)}}\mathrm{}.$$

**Figure 3:**
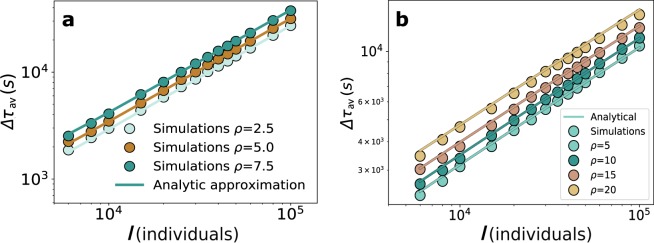
Scaling of the average delay with background in 1D and 2D lattices. Average delay per individual with as a function of the event individuals *I* in (**a**) a 1D lattice and (**b**) a 2D lattice.

In these equations, $L$ is the network length in number of locations. If the value of ${c}_{eff}(x)$ is introduced into Eq. (2) and into the expression for $\Delta {\tau }_{av}$, the simulation results agree with the theoretical predictions as depicted in Fig. 3a. The average delay per individual increases with the intensity of the background $\rho$ due to the reduction of the effective capacity. Moreover, the impact of the background is more pronounced in the center of the lattice than at the extremes. We also corroborate that the background does not affect the scaling exponents.

The same procedure can be taken in higher dimensional networks. The calculation of the load in each location is more involved but still dependent on the link betweenness and, in general, numerically doable. The results for the delay of individuals participating in the event in 2D networks is shown in Fig. 3b. Again the approximation holds well and it is able to estimate the delay for very different values of $\rho$. It is very important to note that even if the values of the delays $\Delta {\tau }_{av}$ do change with the background, the exponent of the scaling with $I$ continues to be the same and equal to the inverse of the lattice dimension.

#### Background individuals

**Figure 4:**
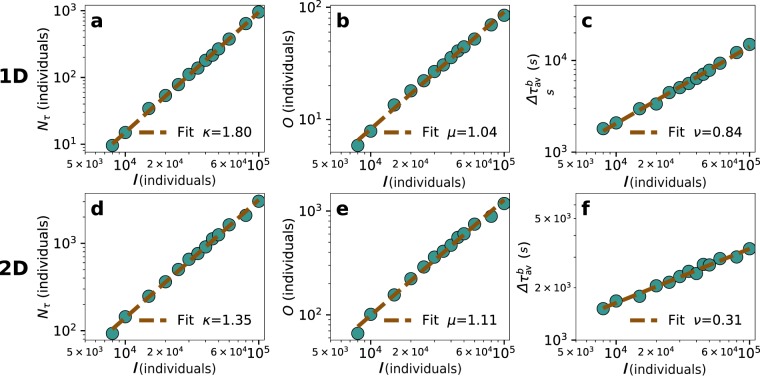
Scaling for the background in regular lattices, the parameters of the simulation are the same as in Fig. 2. In a 1D lattice, scaling with *I* of (**a**) the number of delayed individuals, (**b**) average delay per individual and **c** number of origins affected. In a 2D lattice, scaling with *I* of (**d**) the number of delayed individuals, (**e**) average delay and (**f**) number of origins affected. In the case of the background, only individuals who waited for at least two vehicles were considered as delayed.

The background individuals running through the network also suffer the aftermath of the event. Scaling relations appear with $I$ in the case of the average delay per background individual, the number of delayed individuals and the number of affected trip origins. As before, the exponents are related to the local network dimension. To measure this effect, we propose three metrics: the number of delayed background individuals ${N}_{\tau }$, the number of unique origins of the individuals delayed $O$ and their average delay $\Delta {\tau }_{av}^{b}$. In Fig. 4a–c, we show the scaling of those three metrics with $I$ in 1D lattices. In Fig. 4a, we have the scaling of the total delayed individuals ${N}_{\tau }\sim {I}^{\kappa }$, observing a super-linear scaling with an exponent $\kappa$ close to 2. In this case, we have not been able to find an exact analytical solution, yet the scaling exponent can be explained by a combination of two multiplying factors: (i) the origins of the potential individuals affected are the congested nodes, whose number $(2{r}_{c})$ scales linearly with I; (ii) the time needed by all the nodes to process the queue is proportional to ${r}_{c}$ and, thus, scales as $I$ in 1D (if we assume that all the nodes get congested when the event occurs). As a result, we should expect $\kappa$ to be approximately two. In Fig. 4b, we see a linear scaling of the total number of origins affected $O\sim {I}^{\mu }$, which can be explained by the linear scaling of ${r}_{c}$. The effect is better visible for relatively high values of $\rho$. Finally, in Fig. 4c, we show the scaling of the average delay per delayed individual in the background $\Delta {\tau }_{av}^{b}\sim {I}^{\mu }$. As we could expect, it has a similar exponent to the delay of the individuals participating in the event since they use the same routing protocol, the same network and the only difference lies in the origin of their trips.

We show the scaling of the background in 2D in Fig. 4d–f. In the case of the delayed individuals (Fig. 4d), the exponent can be explained by the same previous argument. In the two dimensional case, the scaling of the number of congested nodes is also linear with $I$, yet the time of the nodes to process the queue grows as ${r}_{c}$, which scales as ${I}^{\frac{1}{2}}$. This implies that $\kappa \approx 1.5$. In Fig. 4e, the number of origins affected scales with $I$, similarly to 1D lattices, as a consequence of the linear growth of the number of congested nodes. Finally in Fig. 4f, the average delay of the individuals in the background displays the same scaling as those in the event.

**Figure 5:**
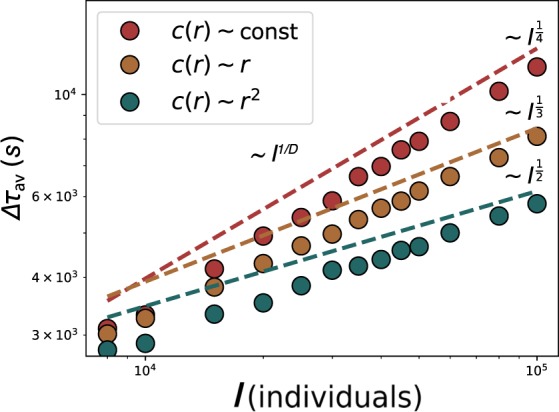
Scaling of $\Delta {\tau }_{av}$ with $I$ in a 2D lattice with a capacity that depends on the distance $r$ from the center of the lattice (event location). Beside the scaling in a lattice with constant capacity (in red), we also explore the delay scaling with $I$ if the transportation capacity available at each node grows linearly $c(r)\sim r$ (in yellow) and in a quadratic way $c(r)\sim {r}^{2}$ (in blue) from the event location. The dashed lines serve as a guide to the eye.

Scaling with heterogeneous capacities: In contrast to regular lattices, real cities display an heterogeneous distribution of capacities and frequencies. Here we investigate if an heterogeneous distribution of capacities can affect the scaling of the average delay. For this purpose, we address the scaling of the average delay imposing a growth of transportation capacity as a function of the distance to the event. We consider three situations: a constant capacity across the network as baseline (the same as above), a linear and a quadratic increase with the distance from the event. Similarly to the previous sections, we calculate the scaling of the average delay $\Delta {\tau }_{av}$ with the event individuals in each of the cases (Fig. 5). The baseline (constant and equal capacity) recovers the previously explained scenario. While the exponents are modified in the other two cases: If the capacity increases linearly with the distance to the event, the scaling approaches 1/3. Otherwise, if it increases quadratically with distance, the scaling of the delay get values closer to 1/4. The increase of capacity with the distance has, therefore, a direct impact on the exponents. This effect is equivalent to have a lattice with a larger local dimension. Therefore, a reformulation of network dimension that takes the scaling of the local capacity around the event into account is needed. To obtain dimensions over 2 in a two dimensional lattice, the capacity has to grow with the distance. In a city, this might relate with having a major transportation hubs nearby, places where the capacity increases by the addition of modes such as metro lines or crossing of several of them.

### Scaling in cities

#### Event individuals

**Figure 6:**
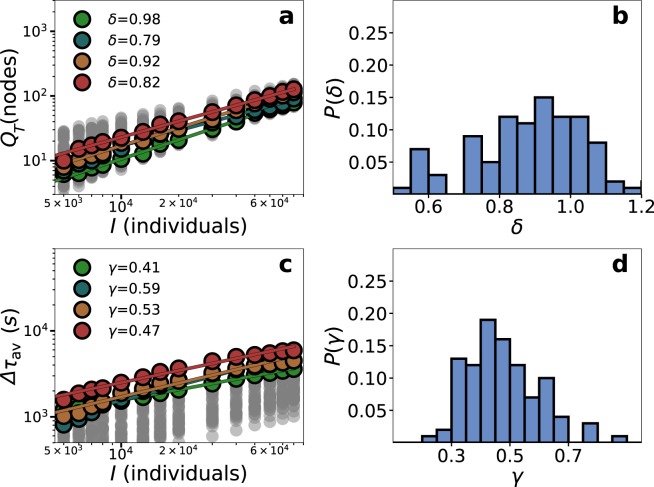
Scaling for the event individuals in Paris. (**a**) Congested nodes as a function of $I$. A stop is considered congested if the queue is higher than its capacity at any moment of the simulations. (**b**) Distribution of the exponents of the scaling of congested nodes. (**c**) Average delay vs $I$. (**d**) Distribution of the exponents of the scaling of the average delay. Simulations are performed in $100$ locations selected at random uniformly within the city boundaries delimited by the transportation networks. In color, we show four locations as highlights and the rest in grey. Simulations were performed with $\rho =8$.

We investigate first how $\Delta {\tau }_{av}$ and ${Q}_{T}$ scale with the number of individuals in the event $I$ for eight empirical public transportation networks. We select in every city $100$ locations at random for probing as event places. Focusing on Paris, the background is set at $\rho =8$ but we will consider later the case without background as well to check the robustness of the results. Starting with ${Q}_{T}$, Fig. 6a displays its scaling ${Q}_{T}\sim {I}^{\delta }$ in four of these locations chosen as illustrative examples of the type of behaviors found. Even though there is only a little more than one decade, the curves in log-log are straight and the scaling exponent is similar across locations. Analyzing the distribution of exponents in more detail (Fig. 6b), we observe that the distribution of $\delta$ is centered around 0.95 and it could be compatible with a linear behavior.

**Figure 7:**
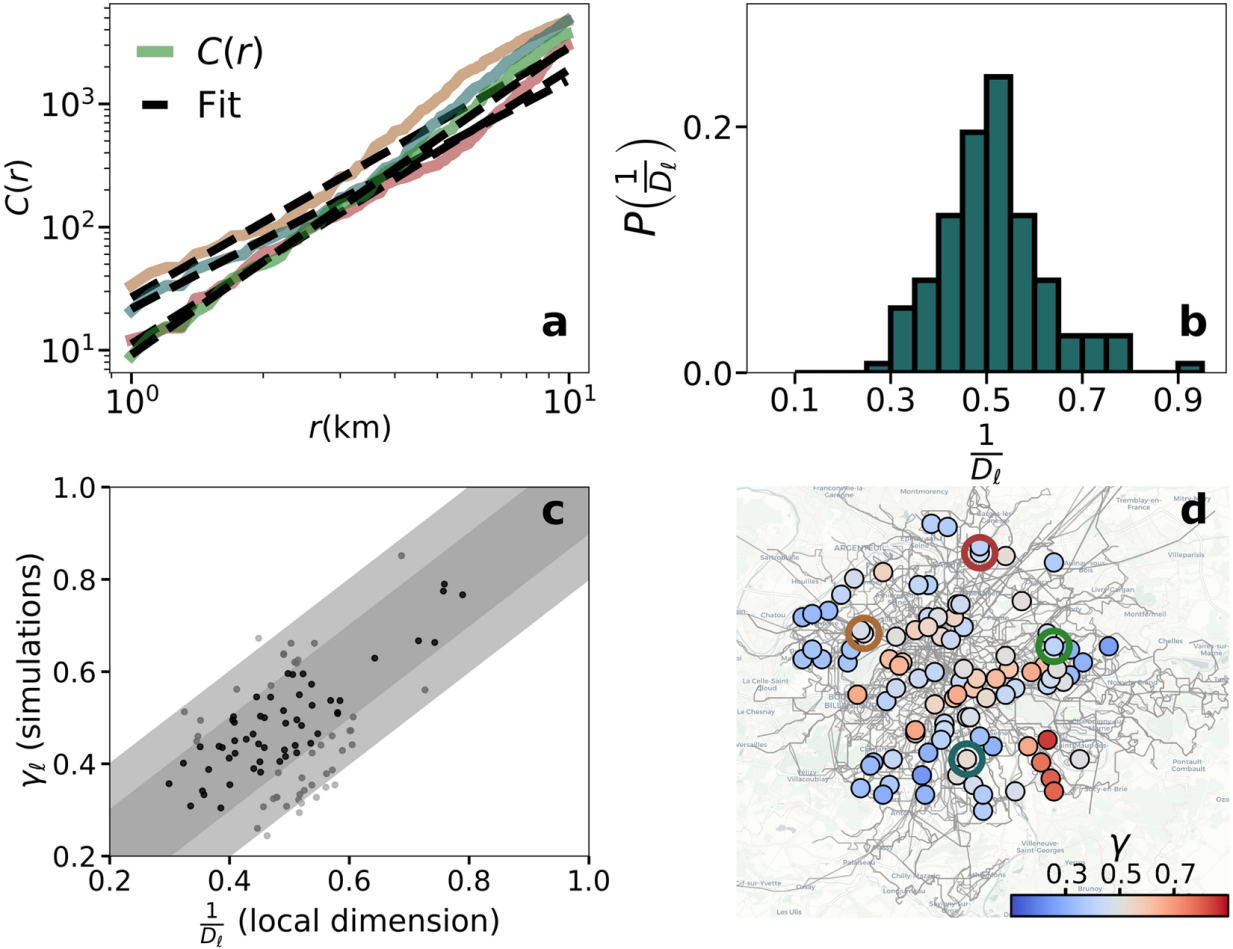
Calculation of the local dimension ${D}_{\ell }$ in the multilayer network of Paris and comparison between ${D}_{\ell }$ and $\Delta {\tau }_{av}$. (**a**) In solid line the scaling of the capacity $C(r)$ as a function of the distance to the event in the same four locations and the power law fit in dashed line. The fit corresponds to the growth of capacity in the empirical network of Paris. (**b**) Distribution of the inverse of the local dimension ${D}_{\ell }$ measured from the growth of capacity in the same 100 locations where simulations were performed (Fig. 6). (**c**) Inverse of the local dimension ${D}_{\ell }$ vs the exponent obtained from the scaling of the average delay with $I$ $({\gamma }_{\ell })$ in the same location $\ell$. The dark and light gray bands correspond to margins of error of 0.1 and 0.2, respectively. (**d**) Map of the scaling exponents of the average delay in the same 100 locations analyzed in Fig. 6. The empty circles in the maps mark the four locations highlighted in color in Fig. 6 and in panel (**a**). The underground map layout is produced using Carto. Map tiles by Carto, under CC BY 3.0. Data by OpenStreetMap, under ODbL.

Looking at the delay of the individuals attending the event, Fig. 6c displays the relation between $\Delta {\tau }_{av}$ and $I$ in the same four locations. As before, the values of the delays depend on the location. The curves show the same order as in Fig. 6a, which means that more congestion implies more delay. The curves can be approximated with scaling relations $\Delta {\tau }_{av}\sim {I}^{\gamma }$, yet the exponents ${\gamma }_{\ell }$ in every location $\ell$ show some variability. In a closer look (Fig. 6d), the exponents are sub-linear in all cases with most of their values between 0.3 and 0.7 and centered around 0.5. As we showed in lattices, the exponents are related to the local characteristics of the network and the inverse of $\gamma$ can be related to a local dimension. The fact that the distribution of ${\gamma }_{\ell }$ is centered around $\mathrm{1/2}$ can be expected from the spatial embedding of the transportation network (See also Supplementary Fig. S2)^47^. The role of the local dimension can be further explored by introducing an alternative metric inspired by^46^ but taking the network capacity into account. As we have shown in Fig. 5, a new formulation of dimension is needed in the presence of heterogeneous capacities. If the total capacity $C(r)$ in persons per second inside a circle of radius $r$ is calculated around the event location $\ell$, we find that ${C}_{\ell }(r)$ grows as ${C}_{\ell }(r)\sim {r}^{{D}_{\ell }}$. The exponent ${D}_{\ell }$ is connected to the number and line types crossing close to $\ell$ and in lattices it corresponds to the network dimension. It is important to stress that ${D}_{\ell }$ is measured in the physical space, not in the network, and we count capacity instead of nodes unlike other metrics of network dimension. An example of how ${D}_{\ell }$ is estimated from the empirical network of Paris is shown in Fig. 7a. If the local dimension is related to the delays produced by the event in the same location, we could expect that ${D}_{\ell }\approx \mathrm{1/}{\gamma }_{\ell }$ in every $\ell$. In fact, the distribution of exponents measured in these two ways are very similar (see Figs. 6d and 7b). The comparison place by place is affected by the noise reigning in numerical simulations for small networks, yet it is in general terms acceptable. As can be seen in Fig. 7c, 55% of the points fall within the dark gray band and 93% within the dark and light bands, which correspond to an error of the exponent of 0.1 and 0.2, respectively. The spatial variability of the scaling obtained in the simulations can be seen in Fig. 7d, where the exponent $\gamma$ has been obtained for the same set of 100 event locations analyzed in Fig. 6. For public transportation systems, the local environment of each location is mostly unique leading to a highly heterogeneous map. The simulations performed contained background in order to provide a more realistic picture, yet the results are similar in the case without it. The absence of background affects the values of the delay but as shown in Supplementary Note “Robustness of the results” and Supplementary Fig. S3 does not alter the local exponent values. As we have seen in lattices, the presence of the background acts by effectively reducing the mode capacity similarly (in percentage) across the full network but it does not modify the scaling with $I$.

**Figure 8:**
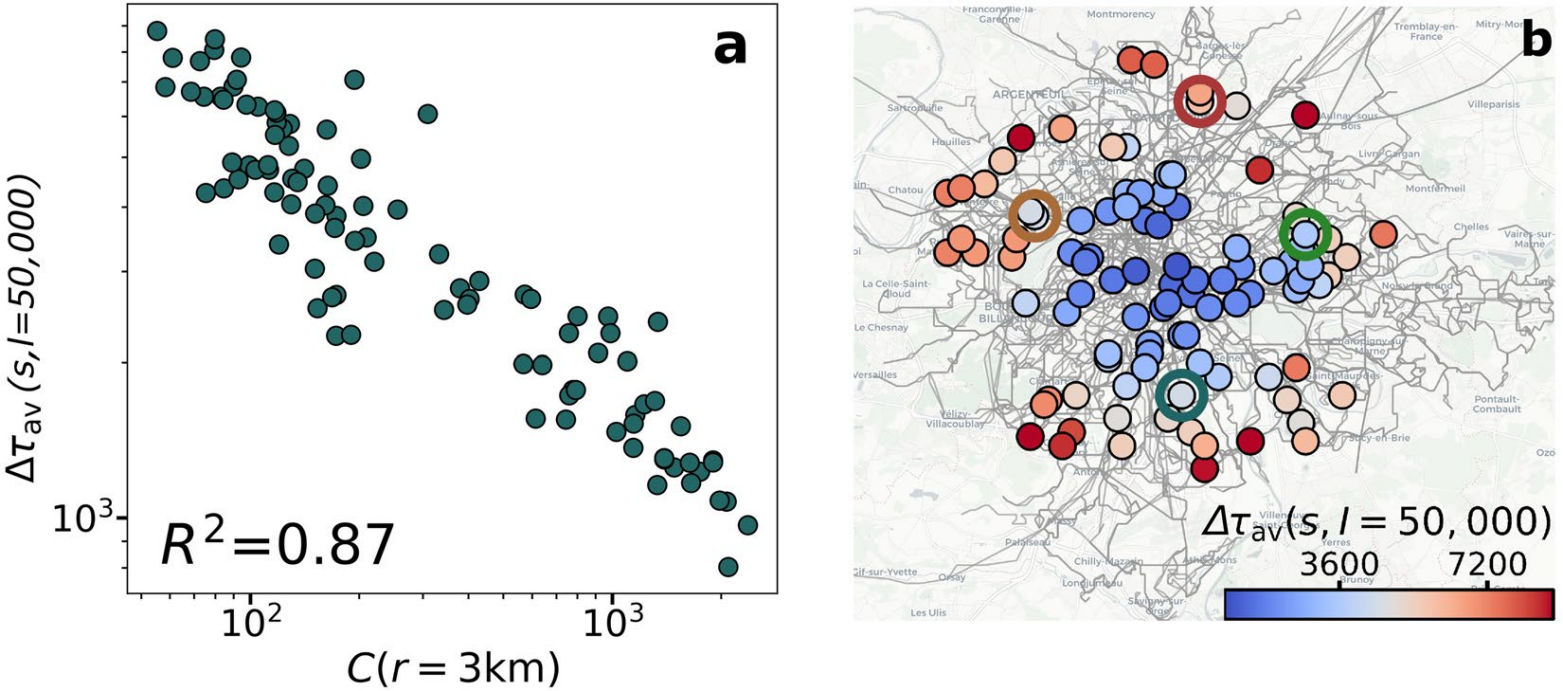
Spatial distribution of delays. (**a**) $\Delta {\tau }_{av}$ for an event of 50,000 individuals as a function of the total capacity within a radius of 3 km. (**b**) Map of the average delay in seconds for an event of 50,000 individuals in the same 100 locations studied in Figs. 6 and 7. The empty circles in the map mark the four locations highlighted in the previous figures. The underground map layout is produced using Carto. Map tiles by Carto, under CC BY 3.0. Data by OpenStreetMap, under ODbL.

While the exponent $\gamma$ controls the growth of $\Delta {\tau }_{av}$ for $I\to \infty$, in real events the value of $I$ can be large but finite. We inspect thus the $\Delta {\tau }_{av}$ for an event gathering $\mathrm{50,000}$ individuals in the previous $100$ locations. As shown in Fig. 8a, the total capacity within a radius of $3$ km from the event location is connected to the value of $\Delta {\tau }_{av}$. The same relation can be observed for the other seven cities (see Supplementary Fig. S9). Note that the spatial distribution of $\Delta {\tau }_{av}$ does not resemble the one of the exponent $\gamma$ (Fig. 8b). It is more important the punctual capacity in the event location than the scaling for large $I$. This leads to a different and stronger spatial pattern in the delay. Locations close to the city center have access to a higher diversity of lines, while in the periphery the service is more limited. As a consequence of the scaling, the radius that provides a better correlation varies with the amount of individuals in the perturbation since more individuals congest a bigger region. Yet, the correlation is always present for many values of the radius (See Supplementary Fig. S4).

Regarding the concrete delays, it is important to note that the maximum values per agent would be attained in case of global network collapse and it is bounded by the difference between walking and transport travel times to the final destination. In practice, in the example for Paris of Fig. 8b, the areas served by high capacity lines (metro) in the center show low delays (below 30 mins), while those in the periphery can go up to two hours and a half. These latter values are a consequence of the lack of infrastructures since such regions are not prepared for massive events and in many cases they have only a few bus lines connecting to the city center. The lines congest under the pressure of the increased demand and this leads to high delays. In a real situation, one could expect a preventive reaction from the transportation managers adding new frequencies and vehicles. This can be easily included in the simulation, helping to better assess the impact of such measures.

Similar results to those shown here for Paris are found in all seven other cities considered in the study (details can be found in Supplementary Note “Results in other cities” and Supplementary Figs. S5–S8).

#### Background individuals

**Figure 9:**
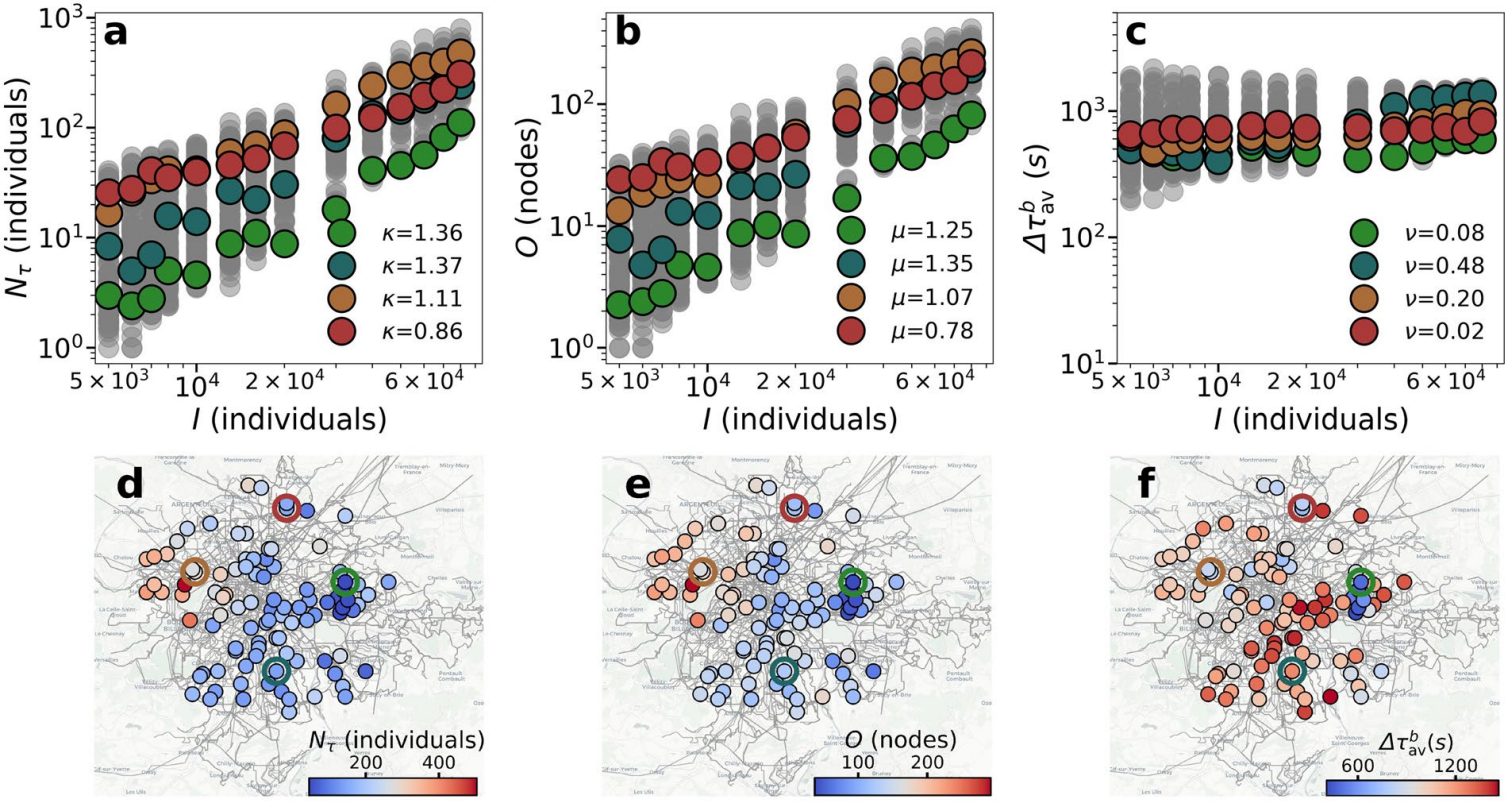
Scaling for the background individuals in Paris. Scaling of the (**a**) delayed individuals, (**b**) origins affected and (**c**) average delay with the number event individuals. Map of the (**d**) delayed individuals, (**e**) origins affected and (**f**) average delay for an event of $\mathrm{50,000}$ individuals. The empty circles in the maps mark the locations of the scaling shown in color in (**a**–**c**). Simulations were performed in the same 100 locations studied in previous figures. The underground map layout is produced using Carto. Map tiles by Carto, under CC BY 3.0. Data by OpenStreetMap, under ODbL.

In Fig. 9a, we show the number of delayed individuals as a function of $I$ with a super-linear scaling relation with an exponent around 1.5. In Fig. 9b, we show the scaling of the number of origins affected, whose exponent falls between 1 and 1.5, yet it depends on the injection rate $\rho$. If $\rho$ is low, the number of congested nodes is low as well. Since the origin of background trips is random, in this regime it may happen that no trip is affected and the number of origins is lower than that of congested nodes. While if $\rho$ is high, all congested nodes are affected origins (scaling linearly with $I$). In Fig. 9c, we have the scaling of the average delay induced to the background individuals that is sub-linear, similarly to the delay of the event individuals. This quantity is more noisy than the two previous metrics due to the small number of delayed individuals and their different origins. The scaling observed resembles the one obtained in 2D lattices, evincing that the mechanisms behind are the same.

Finally, we examine the effect of the event location on the background with a fixed $I=50,000$. In Fig. 9d–f, the number of individuals delayed, their average delay and the number of affected origins are shown in the map. As expected, events located in the periphery impact more individuals and origins, and produce higher delays. Even though the effect is similar as for event participants (Fig. 9f), the locations more affected are different. Event located in the West side of the city seem to affect more origins and individuals than other locations. Similarly, Supplementary Figs. S10–S16 display the results of the scaling for the individuals in the background.

## Discussion

In this work, we studied the recovery of urban transportation systems from massive events. The agglomeration of individuals gives raise to congestion and produces delays in the trips of both individuals participating in the event and those doing their normal trips in the background. We have introduced a model able to simulate the travels across public transportation system featured as multilayer networks. The origin and destination of the agents’ trips are extracted from data in eight worldwide cities. In case of congestion, individuals can reroute their trajectories in an attempt to minimize travel times. We observe a scaling relation between the delay induced to individuals, as well as the number of congested nodes and affected trip origins, with the number of participants in the event $I$.

To gain insights, we have analyzed the case in which the transportation network is a regular lattice. This is a simplistic configuration, unrealistic out of 2D, but it allows for analytical treatment. In fact, we find analytical solutions both in one and two dimensions and an approximate one for dimensions $D > 2$. The average delay per delayed individual and the radius of the congested area both scale as ${I}^{\mathrm{1/}D}$. The number of congested nodes, on the other hand, scales linearly with $I$ independently of the dimension. The individuals in the background are affected by the event too. The number of affected individuals, trip origins and average delay scale with $I$ with different exponents, which can be explained and are related to the lattice dimensionality. Overall, our analytical results in lattices shed light to the mechanisms governing the scaling in transportation networks.

We then simulated massive events in eight global cities, showing that a local dimension must be introduced to explain their scaling. The heterogeneous topology and the multimodal nature of public transportation networks gives rise to a whole range of exponents between 0.3 and 0.7. These values imply that the local dimensionality of some locations in the cities approach 1D, for example terminal areas of the transportation lines where the individuals do not have many choices regarding mobility. On the other hand, there are areas with local dimension larger than 2 as a reflection of the presence of high capacity lines nearby. Furthermore, we introduce an alternative way of measuring the local dimension by studying how the total transportation capacity grows with the distance to the event that show a good agreement with the simulation results. As for lattices, the background mobility also displays a scaling with *I*. Overall, our model allows us to determine the weakest (or strongest) points in a city for the organization of massive events by mapping the local dimension of the network.

## Supplementary information

Supplementary Information.

## Data Availability

In this work, we use two data sources: Geolocated Twitter and transitfeeds.com. For Twitter, the data is downloaded using the streaming API https://developer.twitter.com/en/docs/tweets/filter-realtime/overview. An example of the script employed to obtain geolocated data in a geographical area is provided in the Supplementary Note “Data description”. The data regarding transport networks and their characteristics were download in GTSF format from Transitfeeds web http://transitfeeds.com.
