# Supplementary material for: Scaling in the recovery of urban transportation systems from massive events
Source: Sci Rep. 2020 Feb 17;10:2746. doi: 10.1038/s41598-020-59576-1 (PMC7026446; doi:10.1038/s41598-020-59576-1)
Supplement: Supplementary file 1 — Supplementary Information. [file 41598_2020_59576_MOESM1_ESM.pdf]

Supplementary Information

Scaling in the recovery of urban transportation systems from massive events

Aleix Bassolas, Riccardo Gallotti, Fabio Lamanna, Maxime Lenormand, José J. Ramasco

Table of Contents

Supplementary Note: 1D analytical results **S-2**

Supplementary Note: 2D analytical results **S-8**

Supplementary Note: Scaling with inhomogeneous capacity distribution **S-13**

Supplementary Note: Dataset description **S-14**

Supplementary Note: Robustness of the results **S-15**

Supplementary Note: Results in other cities **S-17**

List of Figures

S1 Average delay as a function of the event location S-7
S2 Cumulative probability distribution of scaling exponents in Paris with real and homogeneous characteristics. S-13
S3 Robustness of the delay and scaling exponents S-16
S4 Robustness of the correlation between the average delay and the total capacity within a radius S-16
S5 Scaling for the event individuals in Amsterdam and Berlin S-17
S6 Scaling for the event individuals in Boston and Madrid S-18
S7 Scaling for the event individuals in Milan and New York City S-18
S8 Scaling for the event individuals in San Francisco S-19
S9 Comparing the local capacity $C(r)$ and the average delay $\Delta\tau_{av}$ S-19
S10 Scaling for the background individuals in Amsterdam S-20
S11 Scaling for the background individuals in Berlin S-20
S12 Scaling for the background individuals in Boston S-21
S13 Scaling for the background individuals in Madrid S-21
S14 Scaling for the background individuals in Milan S-22
S15 Scaling for the background individuals in New York City S-22
S16 Scaling for the background individuals in San Francisco S-23

Supplementary Note: 1D analytical results

In this section, we provide the analytical solution for the scaling of the average delay $\Delta\tau_{av}$ with the number of individuals I in the event for 1D regular lattices. First of all, and independently of the dimension, our system is mainly characterized by two speeds: v_1 for the vehicles and v_2 for the walking layer. The travel time between two nodes in each layer is then calculated by dividing the distance ℓ of a link by the corresponding speeds. Additionally, the transportation lines have two parameters: the capacity c, which is the total number of individuals that fit in a vehicle, and the period of the line f, which is the time elapsed between two consecutive vehicles. The variables of the system are thus:

$$\begin{aligned} v_1 &\ vehicle\ speed; \\ v_2 &\ walking\ speed; \\ c &\ capacity\ of\ the\ vehicles; \\ f &\ period\ of\ the\ line; \\ t_1 &= \frac{\ell}{v_1}; \\ t_2 &= \frac{\ell}{v_2}; \\ &. \end{aligned} \tag{S1}$$

Additionally, if there is background present in the system, ρ individuals will enter the system each second.

We start by calculating the amount of individuals that fit in the queue of a node until walking to the next node becomes preferable. Recall that individuals use optimal paths to estimate travel times and to take routing decisions. In this case, the estimated time to empty a queue q_i observed by individual i is

$$t_{wait,i} = \left(\frac{1}{2} + \left\lceil \frac{q_i}{c} \right\rceil\right) f. \tag{S2}$$

The critical number of individuals that makes walking a better option $q^\star$ is obtained by matching the effective travel time considering congestion $t_1 + t_{wait,i}$ and the walking time between two consecutive stops t_2:

$$t_2 = t_1 + t_{wait,i} = \left(\frac{1}{2} + \left\lceil \frac{q^\star}{c} \right\rceil\right) f. \tag{S3}$$

The expression for $q^\star$ is then given by

$$q^\star = \left\lceil \frac{t_2 - t_1}{f} + \frac{1}{2} \right\rceil c. \tag{S4}$$

The value $q^\star$ captures the number of individuals that will wait at each node, and will be used to calculate the total number of nodes congested.

The individuals in the event are divided between left and right, with a rate that depends on the position of the origin in the lattice. For instance, if the event is located in an extreme, all individuals go towards one side, while if it is located in the middle, the flow splits in two. The number of individuals that go towards each side I_{right} and I_{left} in a lattice of size L and with an event position x, counting locations from 0 to $L-1$, can be calculated as

$$I' = \begin{cases} I_{\text{right}} = \frac{I\,(L-1-x)}{L}, \\ I_{\text{left}} = \frac{I\,x}{L}, \end{cases} \tag{S5}$$

which is a consequence of the uniform distribution of destinations along the space. Considering that each direction can be treated independently, from now on, L' and I' correspond to the length of the segment and the rate of individuals in the chosen direction ($L' = x$ or $L-1-x$, respectively). When the flow of individuals arrive at a node, $q^\star$ of them will stay and the rest of them will walk to the next lattice location. Assuming the conservation of individuals, we can write I' in terms of the people who remain at a node waiting for a vehicle and those that walk all along to their destination. Defining the radius of congestion r'_c as the number of congested nodes in each direction, this expression takes the form

$$\begin{aligned} I' = {} & \overbrace{q^\star}^{\text{node } 0} + \overbrace{q^\star + \frac{I' - q^\star}{L'}}^{\text{node } 1} + \overbrace{q^\star + \frac{I' - 2\,q^\star}{L'} - \frac{I' - q^\star}{L'^2}}^{\text{node } 2} \\ & + \overbrace{q^\star + \frac{I' - 3\,q^\star}{L'} - \frac{2\,I' - 3\,q^\star}{L'^2} + \frac{I' - q^\star}{L'^3}}^{\text{node } 3} + \dots \\ \approx {} & \sum_{\beta=0}^{r'_c-1} \left[q^\star + \sum_{\alpha=1}^{\beta} (-1)^{\alpha+1} \frac{\binom{\beta-1}{\alpha-1} I' - \binom{\beta}{\alpha} q^\star}{L'^\alpha} \right], \end{aligned} \tag{S6}$$

where the first $q^\star$ is the contribution of the queue of the origin node x, the second $q^\star$ is the queue of the first node in the chosen direction and the third term with $1/L'$ corresponds to the number of individuals who arrive by walking and have as destination node $x \pm 1$. The ensuing terms are the contributions of node $x \pm 2$ and $x \pm 3$, etc. The series expansion continues until $r'_c - 1$, after which only $q^\star$ or less individuals remain to allocate. Grouping

terms of the same order in $1/L'$, one can get the following expression for I':

$$\begin{aligned} I' =& r'_c\, q^\star + \frac{(r'_c-1)\, I' - (r'_c\,(r'_c-1)/2)\ q^\star}{L'} \\ &- \frac{((r'_c-1)\,(r'_c-2)/2)\ I' - (r'_c\,(r'_c-1)\,(r'_c-2)/6)\ q^\star}{L'^2} \\ &+ \dots \end{aligned} \tag{S7}$$

The coefficients of each term in I' and $q^\star$ become more complicated as the order in $1/L'$ increases. Still, it is possible to find a closed form for the expansion:

$$I' \approx r'_c\, q^\star + \sum_{\alpha=1}^{r'_c-1} (-1)^{\alpha-1}\, \frac{\binom{r'_c-1}{\alpha}\, I' - \binom{r'_c}{\alpha+1}\, q^\star}{L'^{\alpha}}, \tag{S8}$$

where the symbols $(.)$ are binomial coefficients. Note that we have written the expression as an approximate formula, because after node r'_c, there is a number of individuals (less than $q^\star$) who continue traveling and are not counted in this expression. Using the polynomial expansion and recalling that

$$(1-1/L')^{r'_c-1} = \sum_{i=0}^{r'_c-1} (-1)^i \binom{r'_c-1}{i} \frac{1}{L'^i}, \tag{S9}$$

we can rewrite the sum on the right as

$$I' \approx \left[1 - \left(1 - \frac{1}{L'}\right)^{r'_c-1}\right] I' + \left[1 - \left(1 - \frac{1}{L'}\right)^{r'_c}\right] q^\star\, L'. \tag{S10}$$

This equation can be solved for r'_c as a function of L' and I' yielding

$$r'_c \approx 1 + \frac{\ln\left(\frac{q^\star\, L'}{I' + q^\star\,(L'-1)}\right)}{\ln\left(1 - \frac{1}{L'}\right)}. \tag{S11}$$

The total number of congested nodes will be then given by the sum of the radius of congestion towards both directions $Q_T = r_c(\text{Left}) + r_c(\text{Right})$. In the limit of $L' \to \infty$, the scaling of Q_T with I yields

$$Q_T \sim \frac{I}{q^\star}, \tag{S12}$$

This linear dependency is maintained as long as $Q_T \ll L'$. Otherwise, when $Q_T \to L'$, the whole network is congested, Q_T and the delay saturate with most agents walking to their destination.

From the expression of r'_c, we can estimate the average delay per individual $\Delta\tau_{av}$. First of all, we define the delay as the difference between the real and the expected travel time determined by the optimal path $\Delta\tau = \tau_{real} - \tau_{op}$. In our framework, individuals can suffer the delay in two different ways: either they walk all along until their final destination, or they stay in the queues and wait for their turn to enter into the vehicles. If they walk all the way, the total delay is calculated as the speed difference between walking and using a vehicle multiplied by the number of individuals. For each of the directions, the individuals walking are approximated by the terms displaying powers of $1/L'$ in the expansion of Eq. (S7). By walking i locations at speed v_2, the individuals incur in a delay of $i\,\ell/\Delta v$ (where $\Delta v = v_1 - v_2$). We can write the total delay of walkers as

$$\Delta\tau_{tot,w} \approx \frac{\ell}{\Delta v}\left\{ \overbrace{\frac{I' - q^\star}{L'}}^{\text{node } 1} + \overbrace{2\,\frac{I' - 2\,q^\star}{L'} - 2\,\frac{I' - q^\star}{L'^2}}^{\text{node } 2} \right. \tag{S13}$$

$$+ \overbrace{3\,\frac{I' - 3\,q^\star}{L'} - 3\,\frac{2\,I' - 3\,q^\star}{L'^2} + 3\,\frac{I' - q^\star}{L'^3}}^{\text{node } 3}$$

$$\left. + \dots \right\}.$$

Each term appears multiplied by the position of the node generating it. This expression can be compacted by grouping the terms of the same order in $1/L'$ to obtain

$$\Delta\tau_{tot,w} \approx \frac{\ell}{\Delta v} \sum_{\alpha=1}^{r'_c - 1} \frac{(-1)^{\alpha+1}}{L'^{\alpha}} \left[\frac{(r'_c - \alpha)\,r'_c}{(1+\alpha)} \binom{r'_c - 1}{\alpha - 1} I' - \frac{(r'_c - \alpha)\left((\alpha+1)\,r'_c - 1\right)}{(1+\alpha)\,(2+\alpha)} \binom{r'_c}{\alpha} q^\star \right]. \tag{S14}$$

Suming the terms before I' and $q^\star$ yields

$$\Delta\tau_{tot,w} \approx \frac{\ell}{\Delta v} \left\{ \left[L' - (L' + r'_c - 1)\left(1 - \frac{1}{L'}\right)^{r'_c - 1} \right] I' + \left[\frac{r'_c\,(1 - r'_c)}{2} + L'\,(L' - 1) \right. \right.$$

$$\left. \left. - \; L'\,(L' + r'_c - 1)\left(1 - \frac{1}{L'}\right)^{r'_c} \right] q^\star \right\}. \tag{S15}$$

This equation is valid for each of the directions and the total delay is, thus, the sum of $\Delta\tau_{tot,w} = \Delta\tau_{tot,w}(\text{Right}) + \Delta\tau_{tot,w}(\text{Left})$. While it is important to have an accurate knowledge of the functional form of the delay, the expression for $\Delta\tau_{tot,w}$ in Eq. (S15) tends to zero when $L' \to \infty$ and, therefore, it does not contribute to the scaling of the delay.

The second contribution to the delay corresponds to individuals waiting at queues. The delay suffered by an individual depends on his/her position in the queue. Dividing the $q^\star$ individuals in packs of c, the total delay cumulated in a single node $\Delta\tau_{sn}$ is given by

$$\Delta\tau_{sn} = c\,f \sum_{i=1}^{p-1} i + (q^\star - p\,c)\,p\,f, \tag{S16}$$

where $p = [q^\star/c]$ is the integer part of the ratio. The sum runs over all the packs except the first, which has no delay, and the last, which does not necessarily have c individuals. Solving the sum, we get

$$\Delta\tau_{sn} = f\,p\,\left(\frac{c\,(p-1)}{2} + (q^\star - p\,c)\right). \tag{S17}$$

For the $q^\star$ individuals remaining at each of the queues, we have to add a delay of $f\,q^\star/c$ per link walked. This gives us the total delay of

$$\begin{aligned}\Delta\tau_{tot,v} \approx\ & \overbrace{f\,p\,\left(\frac{c\,(p-1)}{2} + (q^\star - p\,c)\right)}^{\text{node } 0} + \overbrace{\frac{q^{\star 2}}{c}\,f + f\,p\,\left(\frac{c\,(p-1)}{2} + (q^\star - p\,c)\right)}^{\text{node } 1} \\ & + \overbrace{2\,\frac{q^{\star 2}}{c}\,f + f\,p\,\left(\frac{c\,(p-1)}{2} + (q^\star - p\,c)\right)}^{\text{node } 2} + \ldots,\end{aligned} \tag{S18}$$

The previous expression can be reordered to obtain

$$\Delta\tau_{tot,v} \approx r'_c\,f\,p\,\left(\frac{c\,(p-1)}{2} + (q^\star - p\,c)\right) + \frac{q^{\star 2}\,f\,r'_c\,(r'_c - 1)}{2\,c}. \tag{S19}$$

As before, this is the delay in one direction. Therefore, to calculate the average delay per individual $\Delta\tau_{av}$ we need to sum $\Delta\tau_{tot,v}$ in both directions, add it to the one of the walkers $\Delta\tau_{tot,w}$ and divide by the total number of individuals in the event I, yielding

$$\Delta\tau_{av} = \{\Delta\tau_{tot,v}(\text{Right}) + \Delta\tau_{tot,v}(\text{Left}) + \Delta\tau_{tot,w}(\text{Right}) + \Delta\tau_{tot,w}(\text{Left})\}/I.$$

Considering that in the limit $L \to \infty$, $r'_c \sim I'$, $\Delta\tau_{tot,w} \to 0$ and, according to Eq. (S19), $\Delta\tau_{tot,v} \sim I^2$, the scaling of $\Delta\tau_{av}$ with I yields

$$\Delta\tau_{av} \sim \frac{f\,I}{2\,c}. \tag{S20}$$

In finite networks, we can also obtain the number of congested nodes and average delay for any event location. In Fig. S1, we show $\Delta\tau_{av}$ as a function of the position x. Due

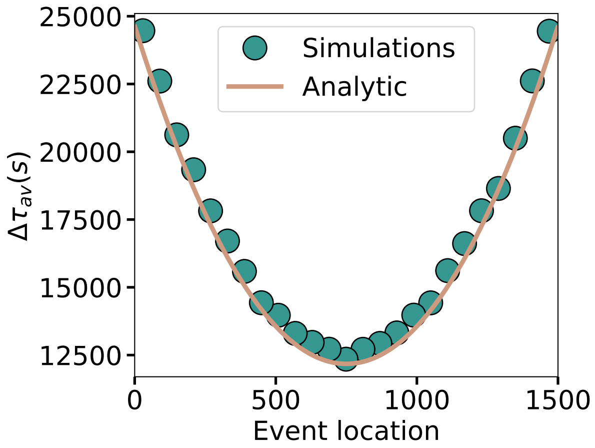

Supplementary Fig. S1: Average delay per individual and its analytical prediction in an 1D lattice with the same event I placed in different lattice locations.

to the split of the stream of individuals, the lowest delay appears for an event in the center of the lattice. Conversely, the highest congestion occurs when the event is introduced in the lattice extremes.

These previous results can be extended to take into account the effect of background individuals. If the background is active, a number ρ of trips will be generated at each time step with an origin and destination selected at random. While conserving the scaling, the background increases the average delay because the new passengers reduce the effective vehicle capacity. We need now to separate the contributions of the individual flows to the left and to the right. The effective capacity for locations to the left of the event can be approximated as

$$c^{l}_{eff}(x) = c - \rho\, f\, \frac{x\,(L-x)}{L\,(L-1)}, \tag{S21}$$

while for those to the right c_{eff} is

$$c^{r}_{eff}(x) = c - \rho\, f\, \frac{(x+1)\,(L-1-x)}{L\,(L-1)}. \tag{S22}$$

Here x refers to the node location, the factors $x\,(L-x)/L\,(L-1)$ and $(x+1)\,(L-1-x)/L\,(L-1)$ are the ratio of shortest paths that go from x to the left and to the right, respectively. They related to the betweenness of x. Eqs. (S21) and (S22) show that the impact of the background is maximum in the central node, while it decreases for the nodes at the extremes. The location of the special event is, therefore, of great relevance since nodes no longer have equal capacity.

To obtain the new expression for the total delay with background, we need to repeat the calculations of Eq. (S18) using $c'_{eff}(x)$ (the one corresponding to the given direction) instead of c. To be more specific, if x is the event location, the $i-th$ congested node to the right and to the left will have an effective capacity $c^{r}_{eff}(x+i)$ and $c^{l}_{eff}(x-i)$, respectively. The sum of the terms of Eq. (S18) in each direction can then be written as

$$\Delta\tau_{tot,\nu} = \sum_{i=0}^{r'_c} f\, p(i) \left(\frac{c\,(p(i)-1)}{2} + (q^\star - p(i)\, c) \right) + \sum_{i=1}^{r'_c-1} i\, \frac{f\, q^{\star 2}}{c'_{eff}(i)}, \tag{S23}$$

where $p(i)$ is the integer part of $[q^\star/c'_{eff}(x \pm i)]$ with the sign and the expression for c_{eff} depending on the direction over the lattice. The integer division in the first summation makes obtaining an analytical expression very difficult. There is, however, an extra approximation that we can take to get an analytical solution for $\Delta\tau_{av}$. This passes through substituting $c'_{eff}(x \pm i)$ by an average value $c'_{eff}(x \pm r'_c/2)$. If we introduce this approximation of c'_{eff} into Eq. (S23), we obtain for each direction

$$\Delta\tau_{tot,\nu} = r'_c\, f\, p \left[\frac{(p-1)\, c'_{eff}(x \pm \frac{r'_c}{2})}{2} + q^\star - p\, c'_{eff}(x \pm \frac{r'_c}{2}) \right] + \frac{q^\star\, f\, r'_c\, (r'_c - 1)}{2\, c'_{eff}(x \pm \frac{r'_c}{2})}, \tag{S24}$$

where $p = [q^\star/c'_{eff}(x \pm r'_c/2)]$. As before, the average delay $\Delta\tau_{av}$ is obtained summing the total delay in both directions and dividing by I.

Supplementary Note: 2D analytical results

In this section, we provide the analytical solution for the scaling in 2D lattices. As detailed in the main manuscript, in two dimensions there are three types of nodes according to the number of suitable directions, and the number of queues at distance r is given by

$$N_q(r) = 4\,\delta_{r,0} + 12\, H_{r,1} + 8\,(r-1)\, H_{r,2}. \tag{S25}$$

where $\delta_{r,0}$ is the Kronecker delta and $H_{r,1}$ is the discrete step function. By dividing the space in four equivalent quadrants and considering only the individuals I' whose destination is within each quadrant, we can write I' as a function of the individuals that stay at a queue, walk all along to their destination and the distance to the closest non congested

location r'_c as

$$I' \approx \overbrace{q^\star}^{\text{distance } 0} + \overbrace{3\,q^\star + \frac{I' - q^\star}{L'_x\,L'_y}}^{\text{distance } 1} + \overbrace{5\,q^\star + \frac{I' - 4\,q^\star}{L'_x\,L'_y} - \frac{I' - q^\star}{(L'_x\,L'_y)^2}}^{\text{distance } 2}$$
$$+ \overbrace{7\,q^\star + \frac{I' - 9\,q^\star}{L'_x\,L'_y} - \frac{2\,I' - 5\,q^\star}{(L'_x\,L'_y)^2} + \frac{I' - q^\star}{(L'_x\,L'_y)^3}}^{\text{distance } 3} + \dots \tag{S26}$$

$$= \sum_{\beta=0}^{r_c-1} \left\{ (2\,\beta + 1)\,q^\star + \sum_{\alpha=1}^{\beta} (-1)^{\alpha+1}\, \frac{\binom{\beta-1}{\alpha-1}\, I' - \frac{2\,\beta-(\alpha-1)}{(\alpha+1)} \binom{\beta}{\alpha}\, q^\star}{(L'_x\,L'_y)^\alpha} \right\},$$

where the lateral size of the lattice is L and (x, y) is the position of the event in the network (allegedly, $(L/2, L/2)$). L'_x (L'_y) is defined depending on the quadrant and the direction of the individuals that are leaving the location of the event. If the movement is towards the right (up), then it is defined as $L'_x = L - x - 1$ ($L'_y = L - y - 1$), otherwise, if it is to the left (down) it is defined as $L'_x = x$ ($L'_y = y$). Likewise 1D lattices, we sum the coefficients appearing in terms of equal power in $(L'_x\,L'_y)$ in Eq. (S26) to obtain

$$I' \approx {r'_c}^2\,q^\star + \sum_{\alpha=1}^{r'_c-1} (-1)^{\alpha+1}\, \frac{\binom{r'_c-1}{\alpha}\, I' - \frac{2\,r'_c-\alpha}{(\alpha+2)} \binom{r'_c}{\alpha+1}\, q^\star}{(L'_x\,L'_y)^\alpha}. \tag{S27}$$

Using the expansion of the binomial $(1 - 1/(L'_x\,L'_y))^\beta$, this expression can be transformed into

$$I' \approx \left[1 - \left(1 - \frac{1}{(L'_x\,L'_y)}\right)^{r'_c-1}\right] I'$$
$$+ q^\star \left[(L'_x\,L'_y) \left(2\,r'_c + 1 - \left(1 - \frac{1}{(L'_x\,L'_y)}\right)^{r'_c}\right)\right.$$
$$\left. -2\,(L'_x\,L'_y)^2 \left(1 - \left(1 - \frac{1}{(L'_x\,L'_y)}\right)^{r'_c}\right)\right]. \tag{S28}$$

Solving the equation in r'_c, we obtain

$$r'_c = -\frac{1}{2} + L' - \frac{\mathrm{Plog}\left((1 - \frac{1}{L'})^{L'-\frac{3}{2}}\,(\frac{q^\star - I'}{2\,L'\,q^\star} + L' - \frac{3}{2})\,\log(1 - \frac{1}{L'})\right)}{\log(1 - \frac{1}{L'})}, \tag{S29}$$

where $\mathrm{Plog}()$ is the product log function and $L' = L'_x\, L'_y$. Asymptotically, in the limit of large lattices $L' \to \infty$, this expression goes with I' (and with I) as

$$r'_c \approx \sqrt{\frac{I'}{q^\star}} \sim \left(\frac{I}{4\, q^\star}\right)^{1/2}. \tag{S30}$$

From r'_c, we can calculate the average delay suffered by the individuals participating in the event. The most straightforward expression is the one concerning the delay of people who arrive at the final destination by walking. These are individuals whose trip destination fall within the congested area, and given the long queues, find more profitable to walk all the way. Their numbers correspond to the terms with the powers in $1/(L'_x\, L'_y)$ in Eq. (S26) and their delay depends on the difference in speed between the vehicles and walking. Recovering the Eq. (S14) for 1D and adapting it to 2D, we get

$$\Delta\tau_{\mathrm{tot},w} = \frac{4\,\ell}{\Delta v}\{ \overbrace{\frac{I' - q^\star}{L'_x\, L'_y}}^{\text{distance 1}} + \overbrace{2\,(\frac{I' - 4\,q^\star}{L'_x\, L'_y} - \frac{I' - q^\star}{(L'_x\, L'_y)^2})}^{\text{distance 2}} + \overbrace{3\,(\frac{I' - 9\,q^\star}{L'_x\, L'_y} - \frac{2\,I' - 5\,q^\star}{(L'_x\, L'_y)^2} + \frac{I' - q^\star}{(L'_x\, L'_y)^3})}^{\text{distance 3}} + \dots$$

$$= \frac{4\,\ell}{\Delta v} \sum_{\alpha=1}^{r_c - 1} \frac{(-1)^{\alpha+1}}{(L'_x\, L'_y)^\alpha} \left\{ \frac{(r_c - \alpha)\, r_c}{1 + \alpha} \binom{r_c - 1}{\alpha - 1} I' \right. \tag{S31}$$

$$\left. - \frac{(r_c - \alpha)\,((2\,\alpha + 4)\; r_c^2 - (\alpha^2 + 2\,\alpha + 3)\, r_c + \alpha - 1)}{(\alpha + 1)\,(\alpha + 2)\,(\alpha + 3)} \binom{r_c}{\alpha} q^\star \right\}.$$

Summing each term of I and $q^\star$, the result is

$$\Delta\tau_{\mathrm{tot},w} = \frac{4\,\ell}{\Delta v} \left\{ \left[L' - (L' + r_c - 1)\, x^{r_c - 1} \right] I' + \left[(4\, L'(6\,(-1 + x^{r_c})\, L'^3\,(12 - 5\, r_c + 12\, r_c^2 + 2\, r_c^3) \right. \right. \tag{S32}$$

$$+ 6\, L'^2\,(18 - 18\, x^{r_c} + 3\,(3 + x^{r_c})\, r_c - 5\,(-2 + 3\, x^{r_c})\, r_c^2 + (3 + 9\, x^{r_c})\, r_c^3 + 2\, x^{r_c} r^4)$$

$$- 3\, L'\,(12 - 12 x^{r_c} - 4\,(-7 + x^{r_c})\, r_c + (17 - 6\, x^{r_c})\, r_c^2 + (-9 + 22\, x^{r_c})\, r_c^3 + 2(-5 + x^{r_c})\, r_c^4 - 2\, r_c^5)$$

$$- r_c(-6 - 29\, r_c - 15\, r_c^2 + (25 + 6\, x^{r_c})\, r_c^3 + 21\, r_c^4 + 4\, r_c^5))$$

$$\left. \left. - r_c^2\,(6 + 11\, r_c + 6\, r_c^2 + r_c^3)\; {}_pF_q(\{2, 2, 2, 1 - r_c\}; \{1, 1, 5\}; 1/L']) \;/(24\, L'\,(1 + r_c)\,(2 + r_c)\,(3 + r_c)) \right] q^\star \right\},$$

where $x = (1 - 1/L')$, ${}_pF_q()$ is the generalized hypergeometric function and $L' = L'_x\,L'_y$. Since this expression is a result of summing a series in terms of $1/L'$, and given that $r_c << L$ its contribution tends to zero in the infinite size limit.

As in any dimension, the most important contribution to the delay comes from the individuals using the vehicles. The calculations are similar to those of 1D, with the additional consideration that the locations have a different number of queues. Given that the delay accumulated while emptying is independent of the dimension (Eq. (S17)), and taking as basis Eq. (S18), the total delay of the individuals using a vehicle in a 2D lattice can be written as

$$\Delta\tau_{tot,v} = \overbrace{4\,f\,p\,\left(\frac{c\,(p-1)}{2} + (q^\star - p\,c)\right)}^{\text{distance } 0} + \overbrace{12\,\frac{q^{\star 2}}{c}\,f + 12\,f\,p\,\left(\frac{c\,(p-1)}{2} + (q^\star - p\,c)\right)}^{\text{distance } 1}$$
$$+ \overbrace{40\,\frac{q^{\star 2}}{c}\,f + 20\,f\,p\,\left(\frac{c\,(p-1)}{2} + (q^\star - p\,c)\right)}^{\text{distance } 2} + \ldots, \tag{S33}$$

where to find the pre-factors for each term one must recall the expression for $N_q(r)$ in Eq. (S25). Again, p is the integer part of $q^\star/c$. Grouping together the different terms of Eq. (S33), one can obtain the expression

$$\Delta\tau_{tot,v} = \frac{q^{\star 2}}{c}\,f\,\sum_{r=1}^{r_c-1} r\,(8\,r+4) + f\,p\,\left(\frac{c\,(p-1)}{2} + (q^\star - p\,c)\right)\sum_{r=0}^{r_c-1}(8\,r+4). \tag{S34}$$

Finally, summing up the series with the number of queues yields

$$\Delta\tau_{tot,v} = \frac{q^{\star 2}}{c}\,f\,\frac{2\,(r_c-1)\,r_c\,(1+4\,r_c)}{3} + f\,p\,\left(\frac{c\,(p-1)}{2} + (q^\star - p\,c)\right)4\,r_c^2. \tag{S35}$$

The asymptotic behavior of $\Delta\tau_{tot,v}$ is obtained by plugging $r_c \sim I^{1/2}$ in Eq. (S35), and yields

$$\Delta\tau_{tot,v} \sim \frac{q^{\star 2}\,f}{c}\,I^{3/2}. \tag{S36}$$

This means, dividing by I, that the average delay per delayed individual scales as

$$\Delta\tau_{av} \sim (q^{\star 2}\,f/c)\,I^{1/2}. \tag{S37}$$

The analytical solution outlined above, as well as the results shown in the main text, hold only when there are no individuals in the background. As in 1D lattices, the introduction of individuals in the background, modifies effectively the capacity of the lines

and we need to find an expression for $c_{eff}(x, y)$. Luckily, the penalty for changing line included in our model simplifies the calculation of the betweenness centrality since there is a maximum of two path alternatives between any pair of nodes. In our framework, the edge betweenness centrality will depend on its location and direction. Therefore, as a function of the lattice side L and the coordinates of the source node (x_0, y_0) and target node (x_1, y_1) of a given edge, can be written as:

$$b((x_0, y_0), (x_1, y_1)) = \frac{\delta_{(y_1-y_0),-1}}{L^2(L^2-1)}\left[(L-y_0)\,y_0 + (L-y_0)\,y_0\,x_0 + (L-y_0)\,y_0\,(L-(x_0+1))\right] \tag{S38}$$

$$+\frac{\delta_{(y_1-y_0),1}}{L^2(L^2-1)}\left[(y_0+1)(L-(y_0+1)) + (y_0+1)(L-(y_0+1))(L-(x_0+1)) + \right.$$

$$\left.(y_0+1)(L-(y_0+1))\,x_0\right] + \frac{\delta_{(x_1-x_0),-1}}{L^2(L^2-1)}\left[(L-x_0)\,x_0 + (L-x_0)\,x_0\,y_0 + \right.$$

$$\left.+(L-x_0)\,x_0\,(L-(y_0+1))\right] + \frac{\delta_{(x_1-x_0),1}}{L^2(L^2-1)}\left[(x_0+1)(L-(x_0+1)) + \right.$$

$$\left.+(x_0+1)(L-(x_0+1))(L-(y_0+1)) + (x_0+1)(L-(x_0+1))\,y_0\right].$$

The average number of individuals of the background going through a link can then be calculated as $\rho\, b((x_0, y_0), (x_1, y_1))\, f$. As an approximation, we will use $c-\rho\, b((\frac{L+r_c(I)}{2}, \frac{L+r_c(I)}{2}), (\frac{L+r_c(I)}{2}+1, \frac{L+r_c(I)}{2}+1))\, f$ as the effective capacity $c_{eff}(L/2, L/2)$.

With the effective capacity, we can obtain an analytical approximation for the average delay with background, yet it requires an extra consideration. In contrast to 1D lattices, it is not enough to replace c by c_{eff}. Before entering a vehicle individuals walking through a link accumulate a delay of $\frac{q^\star}{c_{eff}}$, which corresponds to the time that the previous nodes take to recover. In 2D lattices, it only holds for individuals following the main axes along their route since they need to wait until the previous nodes have emptied. If, instead, they change direction after moving along the main axis, their delay will be different. Effectively they will accumulate $\frac{q^\star}{c}$ in the first walking direction, in which they do not take any vehicle, and $\frac{q^\star}{c_{eff}}$ in the final direction, in which they take a vehicle. In other words, if an individual takes a vehicle upwards in a node at a distance 3 to the right and 1 up of the origin, his delay induced by waiting the vehicle will be given by $f(\frac{q^\star}{c_{eff}} + 3\frac{q^\star}{c})$. Taking this fact into account the total delay of the individuals that wait for a vehicle when there is

background yields

$$\Delta\tau_{tot,v} = (\frac{q^2}{2c_{eff}} + \frac{q^2}{2c})f\frac{8}{3}(r_c - 2)(r_c - 1)r_c$$
$$+ 6(-1 + r_c)r_c f q^2(\frac{1}{3c_eff} + \frac{2}{3c}) + f\,p\left(\frac{c\,(p-1)}{2} + (q^\star - p\,c)\right) 4\,r_c^2. \tag{S39}$$

The first term accounts for the delay of the individuals out of the main axes, whose delay is the mean between $\frac{q^\star}{c_eff}$ and $\frac{q^\star}{c}$. The second term obeys to the delay of the individuals in the main axes, whose delay per link is given by $q^\star(\frac{1}{3c_eff} + \frac{2}{3c})$ which is a consequence of one parallel direction of emptying, which gives $\frac{q^\star}{c_eff}$ and two perpendicular not influenced by the effective capacity, and give $\frac{q^\star}{c}$.

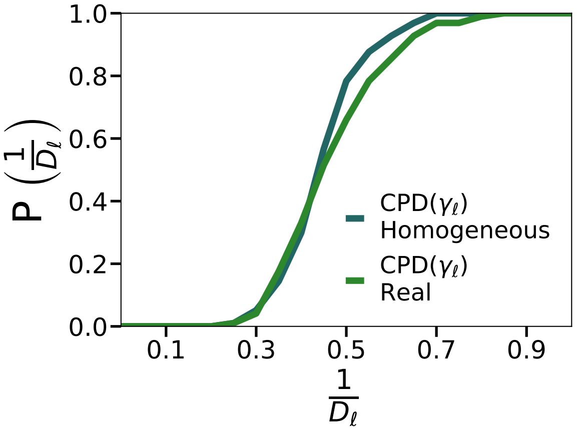

Supplementary Fig. S2: Cumulative probability distribution of scaling exponents in Paris with the real and homogeneous speeds, capacities and periods.

Supplementary Note: Scaling with inhomogeneous capacity distribution

In the main manuscript, we discuss that the broad exponent distribution in cities is a consequence of the different speeds of each transportation modality, the inhomogeneous capacities and frequencies. We performed simulations in Paris keeping all the lines as they are in the map, but all mode speeds to 30km/h, a period of 10 minutes and a capacity of 200 persons. In Fig. S2, we compare the cumulative distribution of exponents for both the

real network and the homogenized one. As can be seen, the distribution of exponents is much more peaked around 0.5 when the characteristics of all lines are homogeneous.

Supplementary Note: Dataset description

The transportation networks studied here were obtained from `https://transitfeeds.com`. They contain the information on the transportation lines in all eight cities, which includes the location of the stops, the departure times and the lapse of times between stops. Table S1 summarizes the transportation network in each city.

City	Transportation nodes	Walking nodes	Modes of transport
Amsterdam	2386	811	3
Berlin	14086	3207	3
Boston	7285	2445	3
Madrid	7815	1634	2
Milan	6886	1919	3
New York City	20289	4207	2
Paris	16503	4921	4
San Francisco	3613	839	3

Supplementary Table S1: Transportation networks dataset description.

The capacities of the vehicles considered are 125 persons for the buses, 250 for tramways, 800 for the subway and 1000 for rail. In the case of San Francisco, the mini metro has a capacity of 125 persons and the cable cars of 70 persons.

To obtain OD matrices we extracted geolocated data from Twitter. A sample of the code used to query Twitter is

```
CONSUMER_KEY = ''
CONSUMER_SECRET = ''
ACCESS_KEY = ''
ACCESS_SECRET = ''
BOX = [x0, y0, x1, y1]
class MyStreamListener(StreamListener):
        def on_status(self, status):
        print(status)
if __name__ == '__main__':
        auth = OAuthHandler(CONSUMER_KEY, CONSUMER_SECRET)
        auth.set_access_token(ACCESS_KEY, ACCESS_SECRET)
        listen = MyStreamListener()
```

```
stream = Stream(auth, listen, gzip=True)
stream.filter(locations=BOX)
```

The details of the data extracted, with the number of users and tweets after the filtering procedure, can be found in Table S2. The temporal window is between 2014 and 2017.

City	Users	Tweets
Amsterdam	20495	1022257
Boston	41799	3471767
Berlin	11209	1371876
Madrid	68794	3643358
Milan	22896	1060747
Paris	49149	6006542
NewYork	227594	19160913
SanFrancisco	44041	1993190

Supplementary Table S2: Twitter dataset description. Number of users and tweets analyzed in each city.

Supplementary Note: Robustness of the results

Here we test the robustness of the results shown in the main manuscript. More concretely we investigate if the background has an effect on the scaling exponents. In Figure S3 (a) we compare the scaling exponents obtained in Paris with and without background. As can be seen, there is a high agreement between them, as the lie near to the diagonal. This results reinforce our analytical solution in lattices, where we have shown that that the background affects the averagedelay but not the scaling. In the case of cities, the background only adds a bit of noise.

In the simulations shown in the main manuscript the penalty for changing line is of 30 seconds. We have performed the same simulations in Paris imposing, instead, a penalty of 120 seconds. As shown in Supplementary Fig.S3(b), the average delay obtained for a penalty of 120 seconds are closely related to those obtained for a penalty 30 seconds, yet, as expected, they are higher.

In Figure 4 of the main manuscript we provide a correlation between the total capacity within a radius of 3 km. The radius which provides a better correlation varies with the individuals in the perturbation. However, these correlations are stable for other values of the radius. In Figure S4 we show how the correlation is still high for different values of the radius.

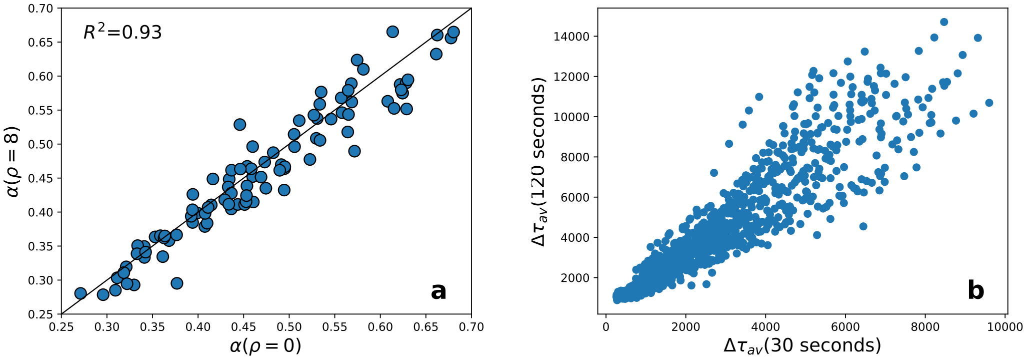

Supplementary Fig. S3: Robustness of the delay and scaling exponents.**a** Comparison of the scaling exponents with ($\rho = 8$) and without background. **b** Comparison between the average delay for a penalty of 30 seconds and of $120 seconds$ for changing line. Each point corresponds to one simulation in one location and one value of I.

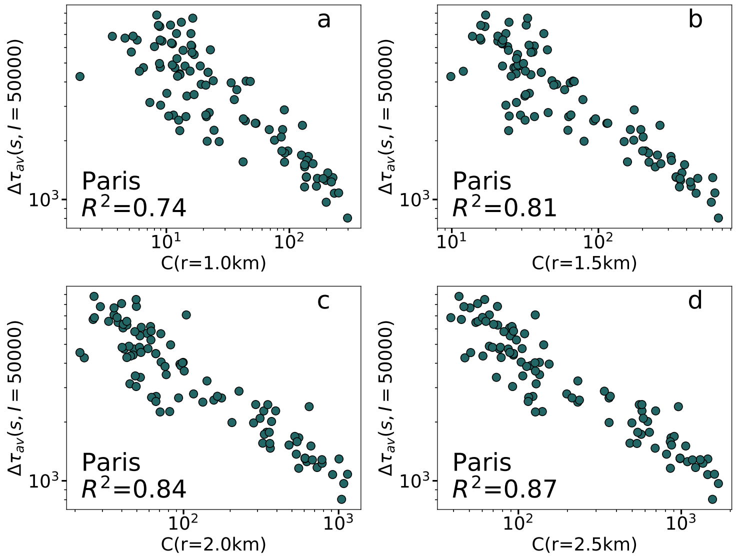

Supplementary Fig. S4: Robustness of the correlation between the average delay and the total capacity within a radius. The values for the radius are **a** 1 km,**b** 1.5 km,**c** 2 km,**d** 2.5 km.

Supplementary Note: Results in other cities

In this section, we provide the scaling results obtained in the other seven cities. Figures S5-S8 depict the results of the scaling for the event individuals in Amsterdam, Berlin, Boston, Madrid, Milan, New York City and San Francisco. Figure S9 provides the correlation between $\Delta\tau_{av}(I)$ for $I = 50,000$ and the total capacity in a radius of 3km for a set of 100 locations in the cities of study. Finally, we examine the effect of the event location on the background, Figures S10-S16 display the results of the scaling for the individuals in the background.

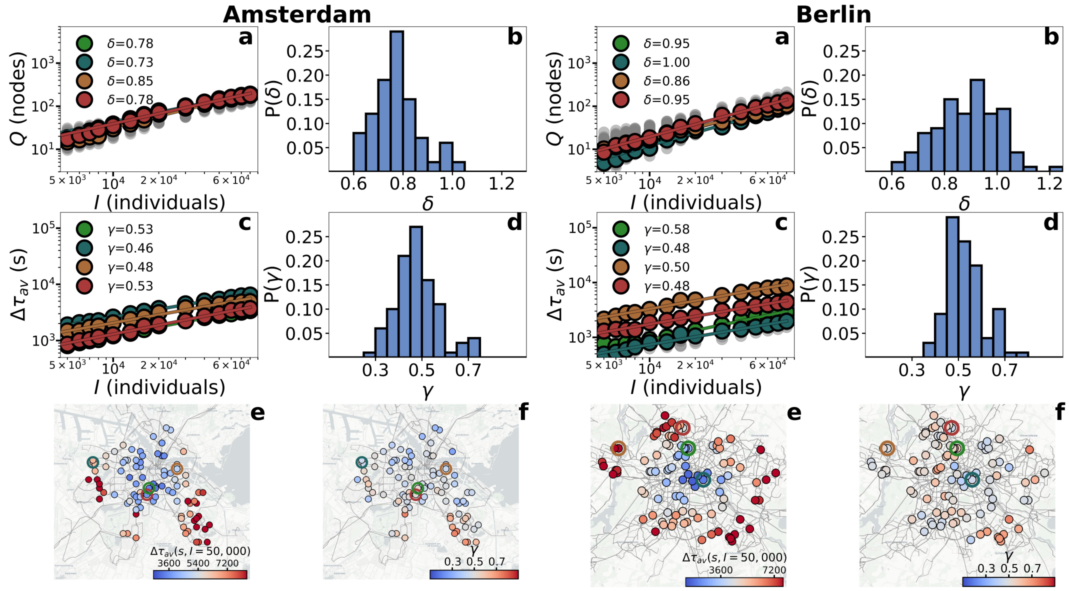

Supplementary Fig. S5: Scaling for the event individuals in Amsterdam and Berlin. **a** Scaling of congested nodes with I. **b** Distribution of the exponents of the scaling of congested nodes. **c** Scaling of the average delay with I. **d** Distribution of the exponents of the scaling of the average delay. **e** Map of the scaling exponents of the average delay. **f** Map of the average delay for an event of 50,000 individuals. The empty circles in the maps mark the locations of the scaling. Simulations were performed with $\rho = 1$ (Amsterdam) and $\rho = 10$ (Berlin).

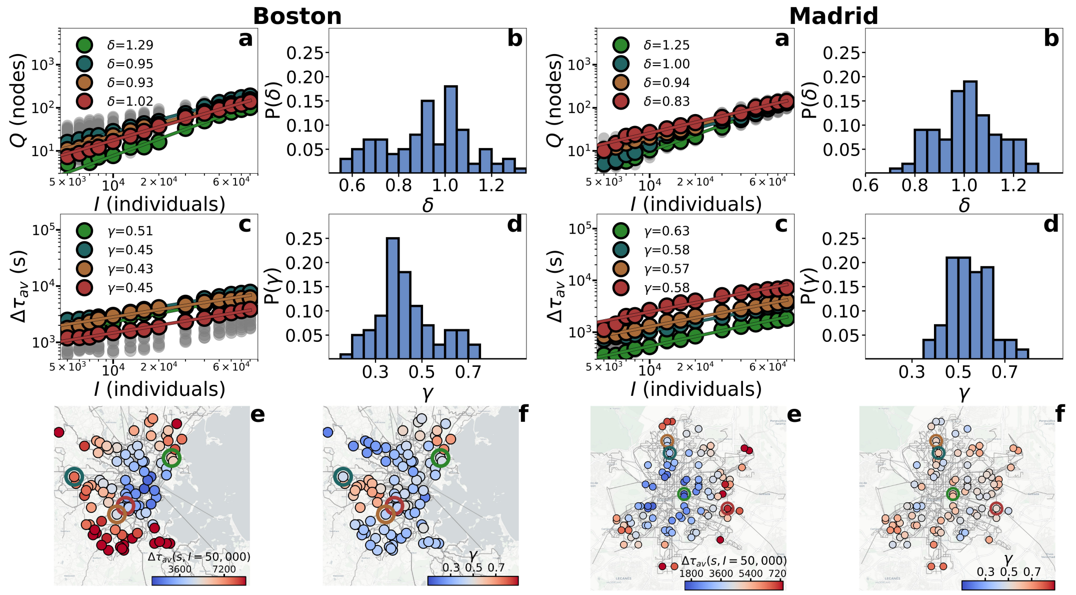

Supplementary Fig. S6: Scaling for the event individuals in Boston and Madrid. **a** Scaling of congested nodes with I. **b** Distribution of the exponents of the scaling of congested nodes. **c** Scaling of the average delay with I. **d** Distribution of the exponents of the scaling of the average delay. **e** Map of the scaling exponents of the average delay. **f** Map of the average delay for an event of $50,000$ individuals. The empty circles in the maps mark the locations of the scaling. Simulations were performed with $\rho = 1$ (Boston) and $\rho = 10$ (Madrid).

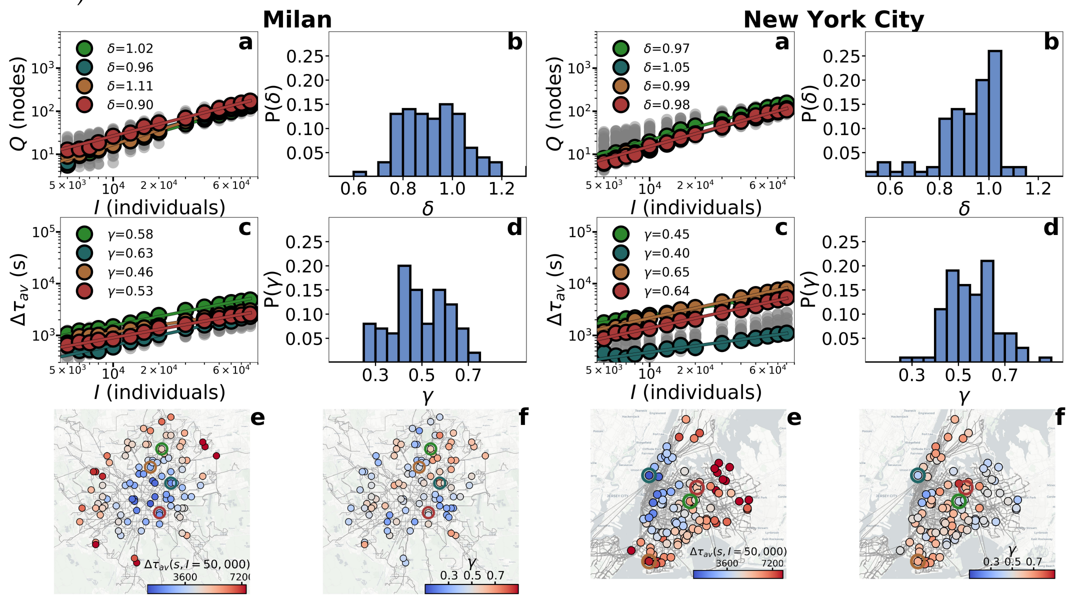

Supplementary Fig. S7: Scaling for the event individuals in Milan and New York City. **a** Scaling of congested nodes with I. **b** Distribution of the exponents of the scaling of congested nodes. **c** Scaling of the average delay with I. **d** Distribution of the exponents of the scaling of the average delay. **e** Map of the scaling exponents of the average delay. **f** Map of the average delay for an event of $50,000$ individuals. The empty circles in the maps mark the locations of the scaling. Simulations were performed with $\rho = 8$ (Milan) and $\rho = 8$ (New York City).

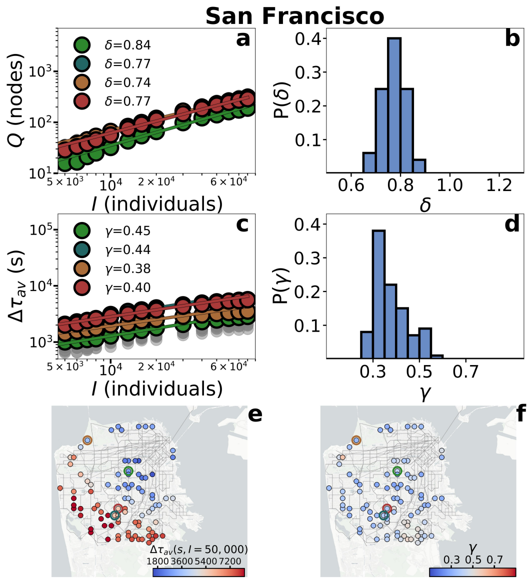

Supplementary Fig. S8: Scaling for the event individuals in San Francisco. **a** Scaling of congested nodes with I. **b** Distribution of the exponents of the scaling of congested nodes. **c** Scaling of the average delay with I. **d** Distribution of the exponents of the scaling of the average delay. **e** Map of the scaling exponents of the average delay. **f** Map of the average delay for an event of 50,000 individuals. The deviation in the exponent distribution from 0.5 is due to the finite size of the area considered. Even the scaling of the variables of the panels (a) and (b) are not of so high quality in this case, with the curves near a plateau for large I. The empty circles in the maps mark the locations of the scaling. Simulations were performed with $\rho = 2$.

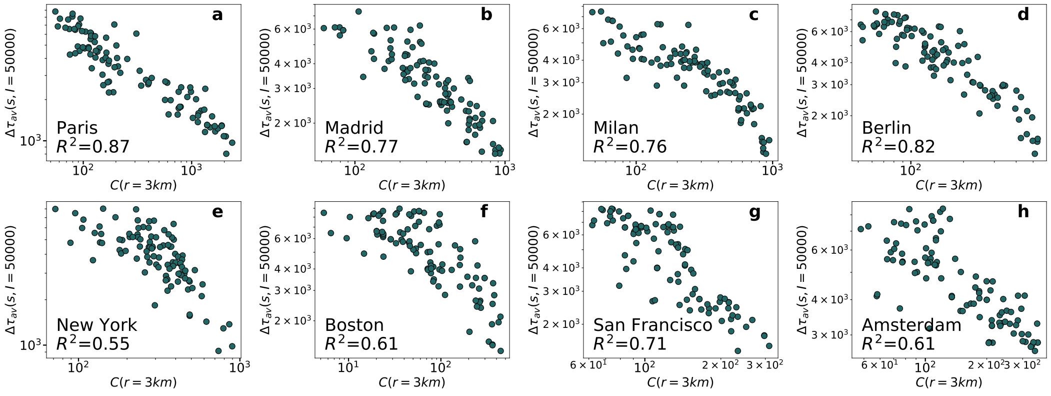

Supplementary Fig. S9: Comparing the total capacity $C(r)$ within a radius of 3 km and the average delay $\Delta\tau_{av}$. $\Delta\tau_{av}$ for an event with 50,000 individuals as a function of the total capacity within a radius of 3 km for the eight cities of study: **a** Paris, **b** Madrid, **c** Milan, **d** Berlin, **e** New York City, **f** Boston, **g** San Francisco, **h** Amsterdam.

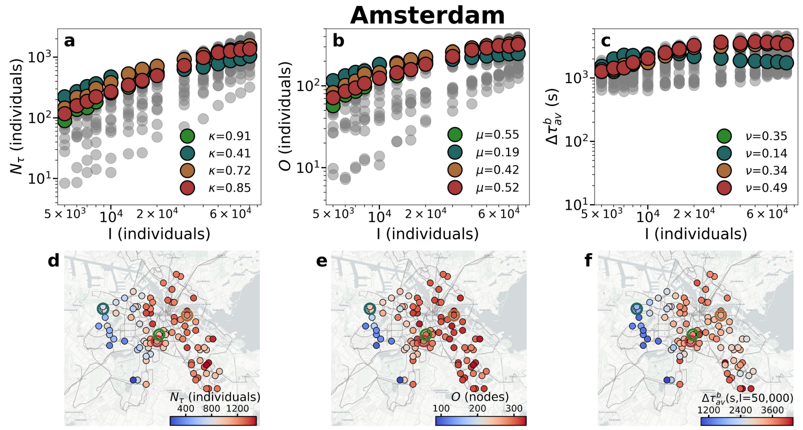

Supplementary Fig. S10: Scaling for the background individuals in Amsterdam. Scaling of the **a** delayed individuals, **b** origins affected and **c** average delay with the number event individuals. Map of the **d** delayed individuals, **e** origins affected and **f** average delay for an event of $50,000$ individuals. The empty circles in the maps mark the locations of the scaling shown in **a**, **b** and **c**. The empty circles in the maps mark the locations of the scaling.

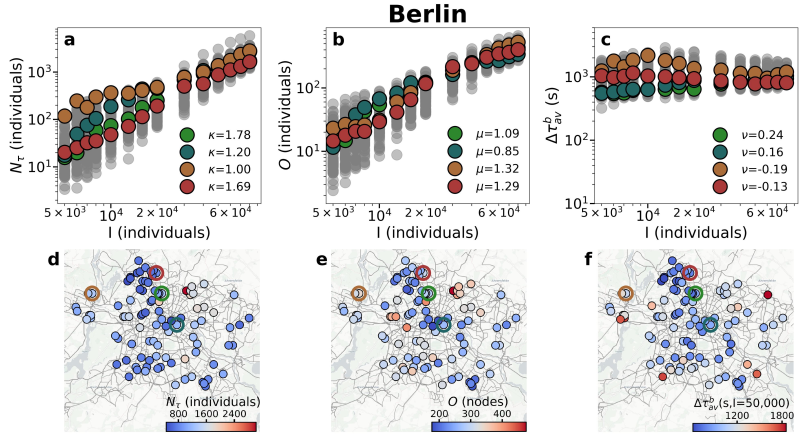

Supplementary Fig. S11: Scaling for the background individuals in Berlin. Scaling of the **a** delayed individuals, **b** origins affected and **c** average delay with the number event individuals. Map of the **d** delayed individuals, **e** origins affected and **f** average delay for an event of $50,000$ individuals. The empty circles in the maps mark the locations of the scaling shown in **a**, **b** and **c**. The empty circles in the maps mark the locations of the scaling.

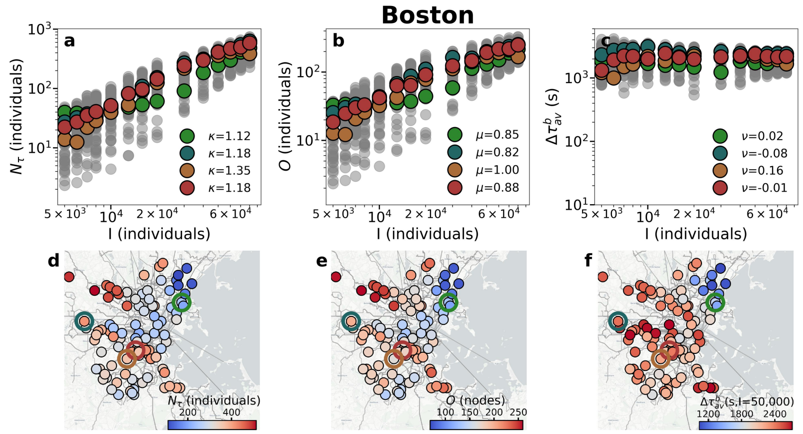

Supplementary Fig. S12: Scaling for the background individuals in Boston. Scaling of the **a** delayed individuals, **b** origins affected and **c** average delay with the number event individuals. Map of the **d** delayed individuals, **e** origins affected and **f** average delay for an event of 50, 000 individuals. The empty circles in the maps mark the locations of the scaling shown in **a**, **b** and **c**. The empty circles in the maps mark the locations of the scaling.

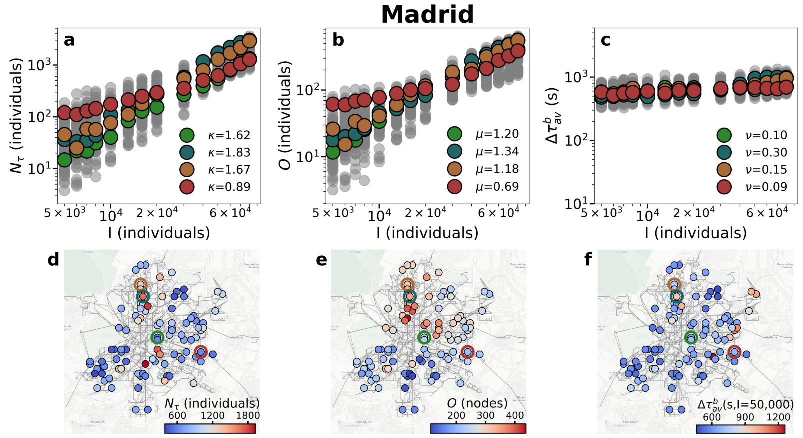

Supplementary Fig. S13: Scaling for the background individuals in Madrid. Scaling of the **a** delayed individuals, **b** origins affected and **c** average delay with the number event individuals. Map of the **d** delayed individuals, **e** origins affected and **f** average delay for an event of 50, 000 individuals. The empty circles in the maps mark the locations of the scaling shown in **a**, **b** and **c**. The empty circles in the maps mark the locations of the scaling.

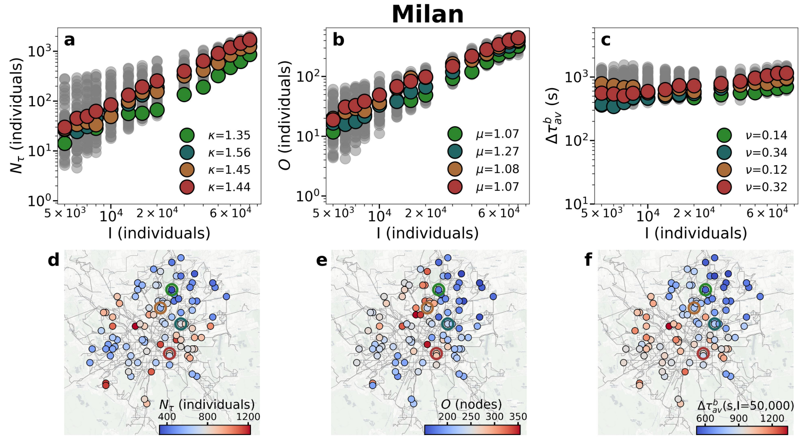

Supplementary Fig. S14: Scaling for the background individuals in Milan. Scaling of the **a** delayed individuals, **b** origins affected and **c** average delay with the number event individuals. Map of the **d** delayed individuals, **e** origins affected and **f** average delay for an event of 50, 000 individuals. The empty circles in the maps mark the locations of the scaling shown in **a**, **b** and **c**. The empty circles in the maps mark the locations of the scaling.

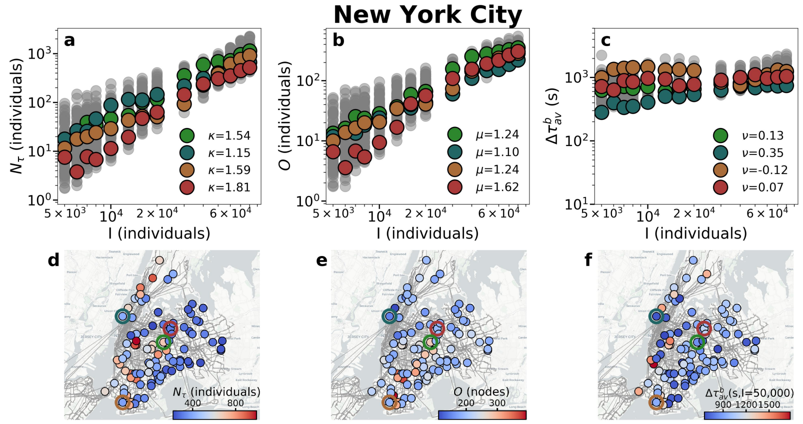

Supplementary Fig. S15: Scaling for the background individuals in New York City.Scaling of the **a** delayed individuals, **b** origins affected and **c** average delay with the number event individuals. Map of the **d** delayed individuals, **e** origins affected and **f** average delay for an event of 50, 000 individuals. The empty circles in the maps mark the locations of the scaling shown in **a**, **b** and **c**. The empty circles in the maps mark the locations of the scaling.

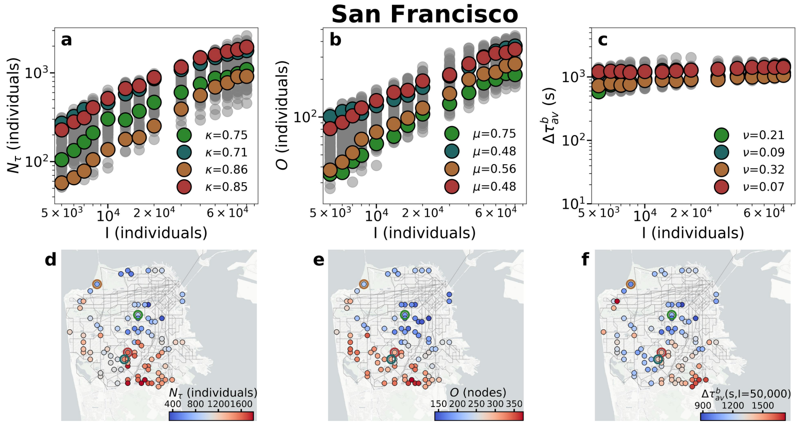

Supplementary Fig. S16: Scaling for the background individuals in San Francisco. Scaling of the **a** delayed individuals, **b** origins affected and **c** average delay with the number event individuals. Map of the **d** delayed individuals, **e** origins affected and **f** average delay for an event of 50,000 individuals. The empty circles in the maps mark the locations of the scaling shown in **a**, **b** and **c**. The empty circles in the maps mark the locations of the scaling.
